# Metabolic reprogramming during hyperammonemia targets mitochondrial function and postmitotic senescence

**DOI:** 10.1172/jci.insight.154089

**Published:** 2021-12-22

**Authors:** Avinash Kumar, Nicole Welch, Saurabh Mishra, Annette Bellar, Rafaella Nasciemento Silva, Ling Li, Shashi Shekhar Singh, Mary Sharkoff, Alexis Kerr, Aruna Kumar Chelluboyina, Jinendiran Sekar, Amy H. Attaway, Charles Hoppel, Belinda Willard, Gangarao Davuluri, Srinivasan Dasarathy

**Affiliations:** 1 Department of Inflammation & Immunity and; 2 Proteomics & Metabolomics Core, Lerner Research Institute, Cleveland Clinic, Cleveland, Ohio, USA.; 3 Department of Pharmacology, School of Medicine, Case Western Reserve University, Cleveland, Ohio, USA.; 4 Department of Integrated Physiology and Molecular Metabolism, Pennington Biomedical Research Center, Baton Rouge, Louisiana, USA.; 5 Department of Gastroenterology, Hepatology & Nutrition, Cleveland Clinic, Cleveland, Ohio, USA.

**Keywords:** Cell Biology, Hepatology, Cellular senescence, Mitochondria, Skeletal muscle

## Abstract

Ammonia is a cytotoxic metabolite with pleiotropic molecular and metabolic effects, including senescence induction. During dysregulated ammonia metabolism, which occurs in chronic diseases, skeletal muscle becomes a major organ for nonhepatocyte ammonia uptake. Muscle ammonia disposal occurs in mitochondria via cataplerosis of critical intermediary metabolite **α**-ketoglutarate, a senescence-ameliorating molecule. Untargeted and mitochondrially targeted data were analyzed by multiomics approaches. These analyses were validated experimentally to dissect the specific mitochondrial oxidative defects and functional consequences, including senescence. Responses to ammonia lowering in myotubes and in hyperammonemic portacaval anastomosis rat muscle were studied. Whole-cell transcriptomics integrated with whole-cell, mitochondrial, and tissue proteomics showed distinct temporal clusters of responses with enrichment of oxidative dysfunction and senescence-related pathways/proteins during hyperammonemia and after ammonia withdrawal. Functional and metabolic studies showed defects in electron transport chain complexes I, III, and IV; loss of supercomplex assembly; decreased ATP synthesis; increased free radical generation with oxidative modification of proteins/lipids; and senescence-associated molecular phenotype–increased **β**-galactosidase activity and expression of p16^INK^, p21, and p53. These perturbations were partially reversed by ammonia lowering. Dysregulated ammonia metabolism caused reversible mitochondrial dysfunction by transcriptional and translational perturbations in multiple pathways with a distinct skeletal muscle senescence-associated molecular phenotype.

## Graphical abstract

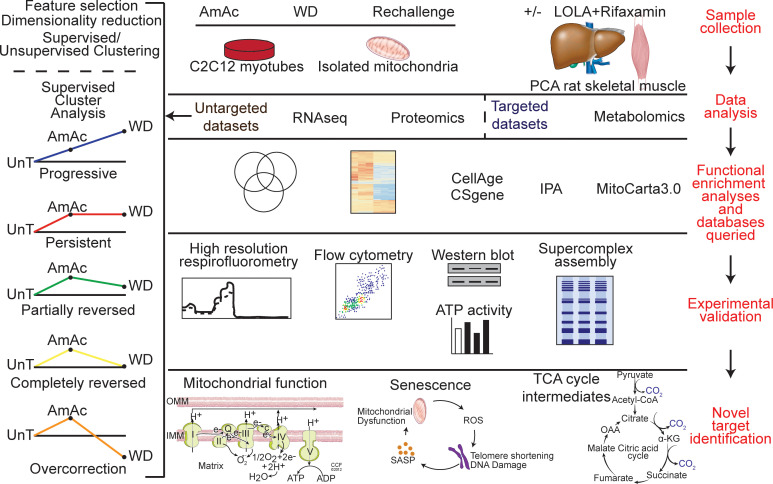

## Introduction

Ammonia is a cytotoxic metabolite generated during the biological processes of amino acid catabolism, purine breakdown, and gut microbial metabolism (1, 2). Physiological ammonia disposal occurs primarily via hepatocyte ureagenesis, with limited renal and pulmonary excretion. However, perturbations in ammonia metabolism and hyperammonemia occur in a number of chronic diseases, including hepatic dysfunction in cirrhosis, heart failure, and chronic obstructive lung disease (3). Increased skeletal muscle ammonia uptake occurs as an adaptive mechanism for nonureagenic ammonia disposal to enhance survival of critical organs (1, 2). In nonureagenic cells, increased uptake and metabolism of ammonia to glutamate and glutamine occurs via cataplerosis or loss of critical tricarboxylic acid (TCA) cycle intermediate, α-ketoglutarate (αKG) (4). Insufficient anaplerotic response with continued cataplerosis results in mitochondrial oxidative dysfunction, decreased ATP synthesis, and increased generation of free radicals with oxidative modifications of proteins and senescence (4–6). Dysregulated cellular protein homeostasis with decreased protein synthesis, one of the most energy-consuming processes within a cell, and increased autophagy, to support critical cellular substrate demands, occur during cellular ATP deficiency and an impaired response to anabolic stimuli that are observed in senescence (7–9).

Previous studies performed in intact myotubes on mitochondrial oxygen consumption during hyperammonemia showed reduced oxygen consumption (4), but defects in the electron transport chain (ETC) components, including their assembly as supercomplexes, and their physiological relevance in vivo were not reported. In the present studies, the mechanistic basis of reduced cellular and tissue mitochondrial oxidative function, ATP synthesis, and intermediary metabolite concentrations during hyperammonemia and whether these changes are reversed by lowering ammonia were evaluated. The translational relevance of these observations is based on data from preclinical models where ammonia-lowering interventions result in reduced tissue concentrations of ammonia and partial restoration of protein homeostasis (10).

Dysregulated protein homeostasis and mitochondrial dysfunction with free radical generation are believed to be causal factors to cellular senescence (9, 11). Accelerated liver senescence and ammonia-induced senescence in astrocytes from patients with liver disease have been reported, but the mechanisms are not known (12, 13). Loss of skeletal muscle mass due to mitochondrial dysfunction and dysregulated protein homeostasis occur during hyperammonemia (4), but whether these abnormalities contribute to the accelerated muscle senescence suggested in liver disease (14) is not known. One potential mechanism is that levels of αKG, which ameliorates senescence (6), are reduced during hyperammonemia (4). Unlike cellular senescence that has been reported primarily in replicating cells, adult skeletal muscle primarily comprises postmitotic cells. Nonreplicative senescence responses, including decreased protein synthesis and senescence-associated secretory phenotype, may occur (15, 16). Even though a phenotype of sarcopenia of aging has been reported in cirrhosis, whether canonical senescence responses occur in the skeletal muscle and if hyperammonemia is a causal factor were evaluated in our models.

Using a complementary multiomics approach integrating whole-cell transcriptomics and proteomics from myotubes and proteomics from isolated mitochondria, we identified temporal clusters of molecular responses. Perturbations in mitochondrial function and oxidative phosphorylation pathway were noted with the greatest reduction in expression of the core and supernumerary subunits of complex I and components of complex IV. Consistently, functional defects in complexes I and IV also were observed in permeabilized myotubes and in skeletal muscle tissue from the hyperammonemic portacaval anastomosis (PCA) rat model. We also observed enrichment of canonical senescence-associated genes in our hyperammonemic models. Experimentally, ammonia disrupted the ETC supercomplexes, and ammonia withdrawal partially restored supercomplex assembly and ETC component function quantified by in-gel activity assays. Ammonia withdrawal in myotubes and ammonia-lowering therapy in the PCA rat restored oxidative phosphorylation and ATP content and decreased free radical generation, with reduced oxidative modification of proteins. Lowering ammonia also restored TCA cycle intermediates in myotubes and skeletal muscle in vivo. Consistent with the increased expression of senescence-associated genes and enrichment of senescence pathways, senescence-associated β-galactosidase activity and staining in myotubes was increased with greater expression of p16^INK^, p21, and phosphorylated p53 during hyperammonemia, and these changes were reversed by ammonia withdrawal/lowering. These data show that hyperammonemia causes reversible perturbations in mitochondrial responses with a senescence-associated molecular phenotype and suggests ammonia lowering is a novel approach to restore cellular homeostasis and potentially reverse senescence.

## Results

### Hyperammonemia causes changes in mitochondrial oxidative components and senescence.

**Figure 1:**
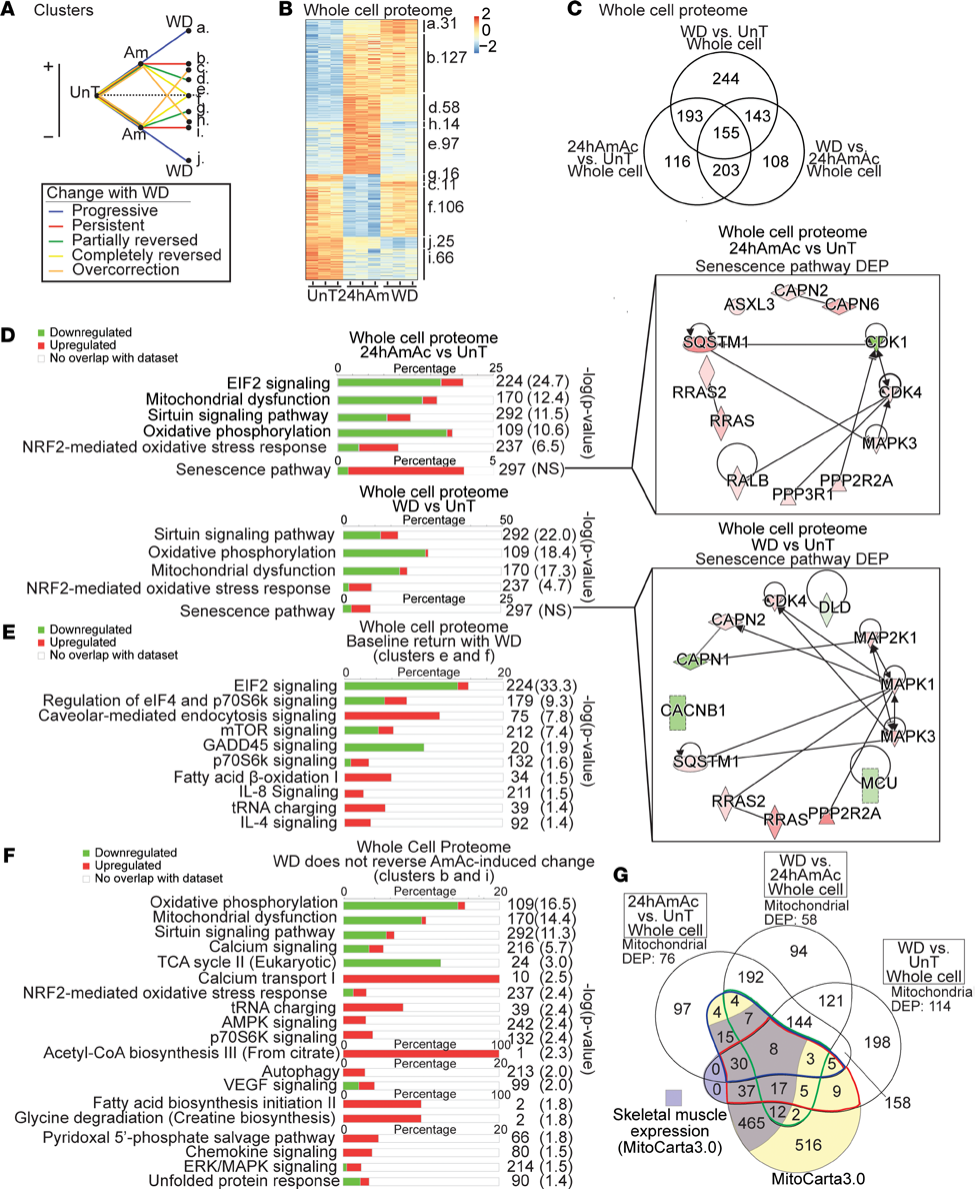
Whole-cell proteomics responses during hyperammonemia and following ammonia withdrawal in differentiated murine C2C12 myotubes. (**A**) Clusters of differentially expressed protein (DEP) responses during hyperammonemia and following ammonia withdrawal (WD). Following a change with hyperammonemia (increase or decrease compared with untreated, UnT), WD results in progressive change in the same direction (compared with UnT controls) (clusters a and j), persistent change (b and i), partial reversal (d and g) or complete reversal to baseline (e and f), or overcorrection (c and h). (**B**) Heatmap of DEPs from whole-cell proteome from UnT, those treated with 24 hours ammonium acetate (24hAmAc), or after WD and the number of DEPs in each cluster. (**C**) Venn diagram of unique/shared DEPs (UnT, 24hAmAc, and WD). (**D**) Functional enrichment analysis of DEPs (24hAmAc vs. UnT) and changes that persist despite ammonia withdrawal (WD vs. UnT) with the network of DEPs within the senescence pathway. (**E**) Most enriched canonical pathways in the “completely reversed” clusters (e and f). that changes with 24hAmAc treatment but returns to baseline (UnT) with the network of DEPs within the senescence pathway. (**F**) Most enriched canonical pathways in the “persistent” clusters (b and i) that change with 24hAmAc and do not improve with WD. (**G**) Venn diagram of unique/shared DEPs in the myotube proteome data set overlaid with the genes in MitoCarta3.0 (yellow) and skeletal muscle expressed DEPs within MitoCarta3.0 (purple). Verified mitochondrial DEPs in the WD versus UnT comparison (red line), WD versus 24hAmAc (green line), and 24hAmAc versus UnT (blue line). Mitochondrial DEP numbers refer to those matched with MitoCarta3.0. All cellular experiments were done in *n* = 3 biological replicates. *P* value cutoff for DEPs was set at *P* < 0.05 using an unpaired 2-tailed Student’s *t* test. For **D**–**F**: green, decreased expression; red, increased expression. Significance cutoff for all pathways is –log(*P* value) ≥ 1.3 by a right-sided Fisher’s exact test. NS, not significantly enriched.

Insights into ammonia-induced perturbations in mitochondrial function and consequent cellular responses were obtained by multiomics analyses. Whole-cell transcriptomics were integrated with proteomics from mitochondria isolated from hyperammonemic myotubes and from gastrocnemius muscle from the PCA/sham rats and myotubes in response to ammonia withdrawal/lowering. The transcriptomic and proteomic responses in the untreated (UnT) control and hyperammonemic myotubes and in those that underwent ammonia withdrawal showed distinct temporal clusters of responses. Temporal cluster analyses have been reported by us earlier and are described in the Methods section. These clusters were then grouped based on the similarity of the direction of responses during hyperammonemia and following ammonia withdrawal compared with UnT myotubes (Figure 1A). We show a heatmap and a Venn diagram of DEPs identified in the whole-cell proteome from UnT and ammonia-treated myotubes and from those after ammonia withdrawal and the number of DEPs within each cluster (Figure 1, B and C). Mitochondrial functions including oxidative phosphorylation, nuclear factor erythroid-2 related factor 2–mediated (NRF2-mediated) oxidative stress response, senescence, and sirtuin signaling pathways were among those most enriched in our whole-cell proteome data set during hyperammonemia and following withdrawal (Figure 1D). These data show that mitochondrial pathway dysfunction during hyperammonemia was partially reversed following ammonia withdrawal. The senescence pathway networks of DEPs at 24 hours and following ammonia withdrawal showed that while the senescence pathway was not significantly enriched, the majority of the senescence-associated DEPs were increased. Ammonia withdrawal resulted in a reduction in a number of senescence-related DEPs. The clusters with the highest numbers of DEPs were those that had expression levels that significantly changed with hyperammonemia but returned to baseline (UnT) expression levels after ammonia withdrawal, i.e., “completely reversed” group, and the group of DEPs that had a persistent change in expression after hyperammonemia despite withdrawal, i.e., “persistent” group. Functional enrichment analysis performed on the temporal clusters showed that DEPs that were altered (increased/decreased) in response to hyperammonemia and completely reversed (clusters e and f) from those in UnT myotubes were those involved in protein synthesis (Figure 1E). DEPs that changed (increased/decreased) during hyperammonemia but did not reverse with ammonia withdrawal (clusters b and i) were primarily regulators of mitochondrial oxidative function (Figure 1F). The DEPs during hyperammonemia that had continued change in the same direction following ammonia withdrawal compared with UnT controls (clusters a and j) primarily regulated metabolic pathways and calcium signaling; the DEPs that changed with ammonia treatment and had partial but incomplete reversal with withdrawal (clusters d and g) regulated neuronal generation/degeneration and receptor protein-tyrosine kinase, α-adrenergic, and G protein–coupled receptor signaling; and the DEPs with expression levels that changed from baseline (increased/decreased) with hyperammonemia, but then had an opposite change in expression with withdrawal (clusters c and h), regulated phagocytosis and endocytosis (Supplemental Table 1; supplemental material available online with this article; https://doi.org/10.1172/jci.insight.154089DS1). We then aligned the DEPs in the whole-cell proteomics and MitoCarta3.0 (a database of validated mouse and human proteins that localize to the mitochondria) (17) to identify unique and shared mitochondrial localized proteins (Figure 1G). Since the mitochondrial fraction was part of the whole-cell proteome, a number of proteins were shared with MitoCarta3.0. Skeletal muscle–specific molecules in MitoCarta3.0 also matched with DEPs in our whole-cell myotube proteome, demonstrating that our proteomics analyses identified altered expression of a number of known muscle mitochondrial proteins.

**Figure 2:**
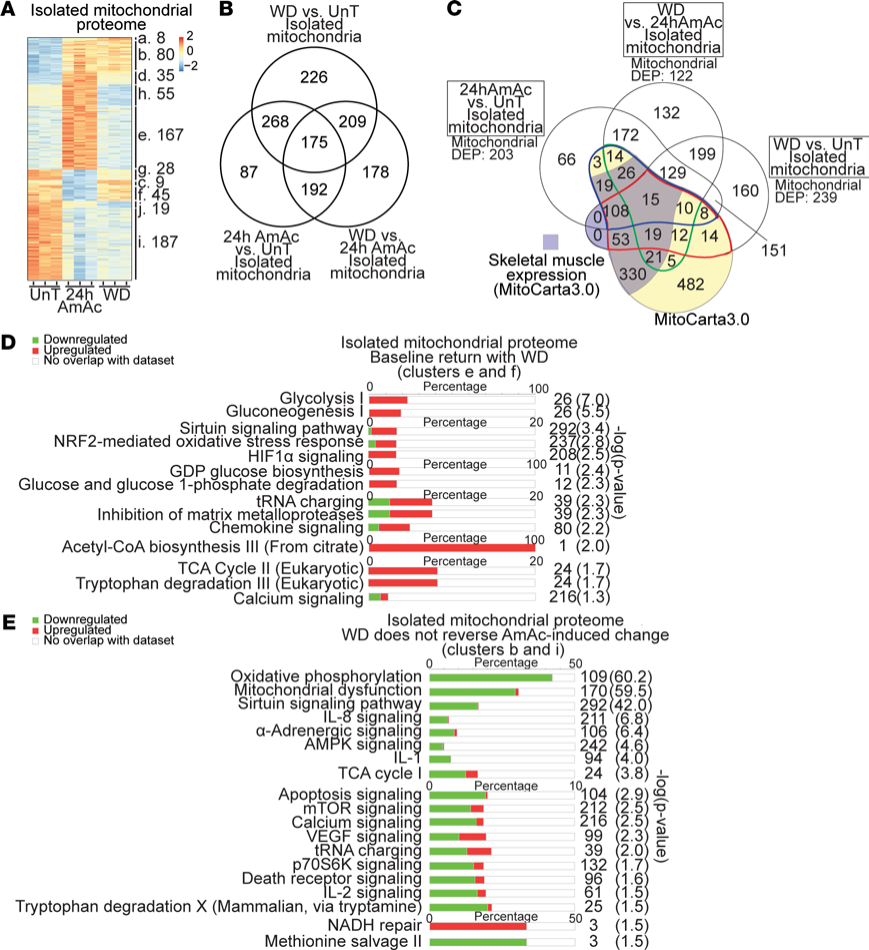
Proteomics from mitochondria isolated from myotubes during hyperammonemia and following ammonia withdrawal. (**A**) Heatmap of differentially expressed proteins (DEPs) from mitochondria isolated from untreated (UnT), treated with 24h ammonium acetate (24hAmAc), or 24 hours of AmAc withdrawal (WD) C2C12 myotubes arranged by clusters that shows the number of DEPs within each cluster. (**B**) Venn diagram showing unique and shared DEPs in isolated mitochondria from UnT, 24hAmAc, or WD treated C2C12 myotubes. (**C**) Venn diagram of unique and shared DEPs from mitochondria isolated from C2C12 myotubes that were UnT, or treated with 24hAmAc or WD, overlaid with the genes listed in MitoCarta3.0 (shaded in yellow) and the DEPs known to be expressed in skeletal muscle within MitoCarta3.0 (shaded in purple). Verified mitochondrial DEPs from MitoCarta3.0 in the WD versus UnT comparison (enclosed by a red line), WD versus 24hAmAc (green line), and 24hAmAc versus UnT (blue line). Mitochondrial DEPs numbers refer to those matched with MitoCarta3.0. (**D**) Most enriched canonical pathways in the proteome from isolated mitochondria in the “completely reversed” cluster that change (clusters e and f) with 24hAmAc but return to baseline (UnT). (**E**) Most enriched canonical pathways in the proteome from isolated mitochondria in the “persistent” clusters (b and i) that change with 24hAmAc and do not improve with WD. All cellular experiments were done in *n* = 3 biological replicates. *P* value cutoff for differentially expressed proteins was set at *P* < 0.05 using an unpaired 2-tailed Student’s *t* test. (**D** and **E**) Green, decreased expression; red, increased expression. Significance cutoff for all pathways was set at –log(*P* value) ≥ 1.3 by the right-tailed Fisher’s exact test.

**Figure 3:**
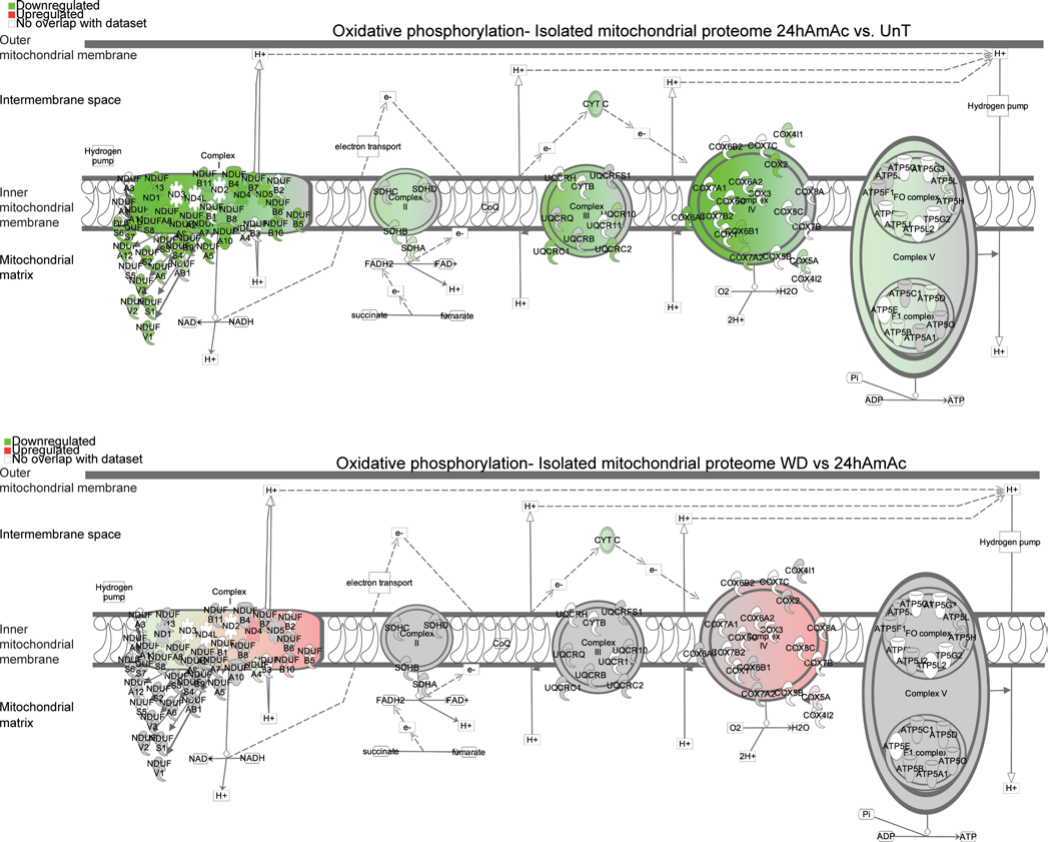
Oxidative phosphorylation pathways during hyperammonemia and following ammonia withdrawal. Differentially expressed electron transport chain (ETC) subcomponents during hyperammonemia and following ammonia withdrawal (WD). Pathways of ETC components at 24hAmAc (vs. UnT) and following WD (vs. 24hAmAc) in the mitochondrial proteome. All cellular experiments were done in *n* = 3 biological replicates. *P* value cutoff for differentially expressed proteins was set at *P* < 0.05 using an unpaired 2-tailed Student’s *t* test. Green = decreased expression, red = increased expression. Significance cutoff for all pathways was set at –log(*P* value) ≥ 1.3 by a right-tailed Fisher’s exact test.

Proteomics analyses from mitochondria isolated from myotubes showed significant differences in the 3 treatment groups of myotubes arranged by clusters (Figure 2A) with shared and unique proteins found in the 3 groups (Figure 2B). We also identified a number of molecules in isolated mitochondria that are currently believed to be nonmitochondrial proteins and need to be verified in future studies that may help update MitoCarta3.0. Also, interestingly, a number of the DEPs in our mitochondrial proteome were not listed in the MitoCarta3.0 database (Figure 2C). Potential explanations for these observations include mitochondrial transport of nonmitochondrial proteins, interaction between mitochondria and other organelles, or proteins not yet listed on MitoCarta3. Even though contamination of the mitochondrial fraction with other cellular fractions is a possibility, immunoblots of our mitochondrial fractions showed no nuclear, cytosolic, or endoplasmic reticulum contamination, demonstrating the purity of the isolated mitochondria (Supplemental Figure 1). Our identification of known cytosolic molecules/pathways in the isolated mitochondrial proteomic data set suggests that sensitive mass spectrometric analyses can detect low levels of expression of these molecules or that these proteins could not be identified on immunoblots since the current antibodies do not detect those epitopes identified on proteomics. Our observations also suggest that validation of the highly expressed proteins in isolated mitochondria that were not listed on MitoCarta3.0 will need evaluation and confirmation that they are authentic mitochondrial proteins. We next performed functional enrichment analysis using all DEPs from isolated mitochondria in the “completely reversed” with withdrawal and the “persistent” change with withdrawal clusters. Both clusters had significant enrichment of mitochondrial oxidative function and regulatory pathways of protein homeostasis (Figure 2, D and E, and Supplemental Table 2). Interestingly, not all molecules/pathway components that were altered during hyperammonemia recovered after ammonia withdrawal. Specifically, in the proteome from isolated mitochondria, hyperammonemia resulted in a variable decrease in the expression of components from all 5 ETC complexes, with the greatest reduction in expressions occurring in complexes I and IV followed by that in complex III (Figure 3). These changes were only partially restored with ammonia withdrawal, including those components on complex I involved in the electron transfer to the ubiquinone (Figure 3 and Supplemental Figure 2). Expression of both nuclear and mitochondrially encoded components of complex I were decreased, and analyses of the modules N (electron input from NADH), Q (delivery of electron to ubiquinone), and P (proton translocation) showed no specific pattern of recovery after ammonia withdrawal. Similarly, changes in components of complexes III and IV also did not show preferential reduction in expression of nuclear or mitochondrially encoded DEPs or those involved in the electron acceptor or donor components (Supplemental Table 3).

**Figure 4:**
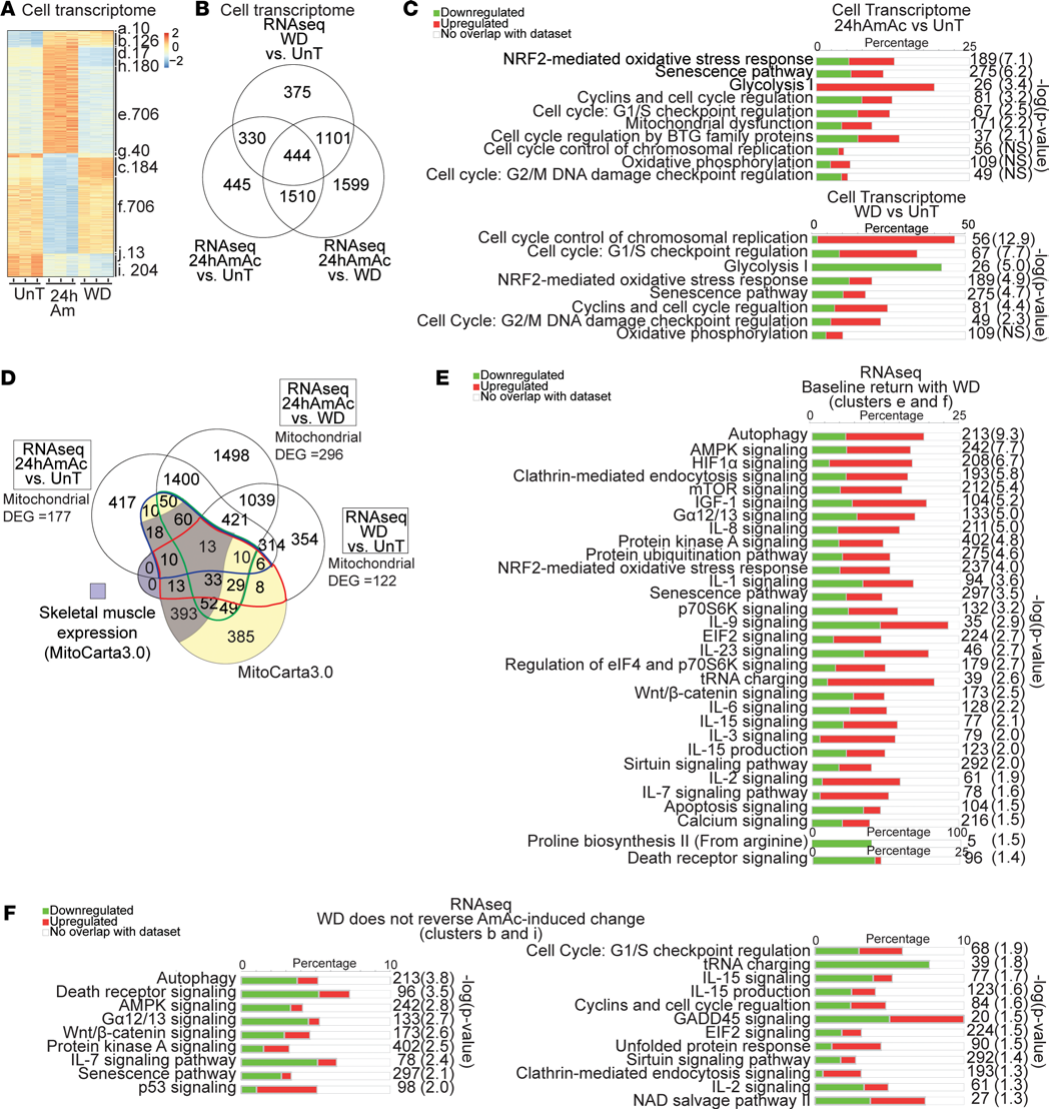
Whole-cell transcriptomics from hyperammonemic myotubes show changes in the mitochondrial and senescence pathways. (**A**) Heatmap of differentially expressed genes (DEGs) in untreated (UnT), 24 hours ammonium acetate (24hAmAc) treated, or following 24 hours of AmAc withdrawal (WD) in C2C12 myotubes arranged by clusters with the number of DEGs indicated. (**B**) Venn diagram of unique and shared DEGs in the cellular transcriptome with UnT, 24hAmAc, and WD comparisons. (**C**) Functional enrichment analysis of DEGs during hyperammonemia (24hAmAc vs. UnT) and changes that persist despite ammonia withdrawal (WD vs. UnT comparison). (**D**) Venn diagram of unique and shared DEGs overlaid with the genes listed in the MitoCarta3.0 (shaded in yellow) and the DEGs known to be expressed in skeletal muscle within the MitoCarta3.0 (shaded in purple). Verified mitochondrial DEGs in the WD versus UnT comparison (enclosed by a red line), WD versus 24hAmAc (green line), 24hAmAc versus UnT (blue line). Mitochondrial DEGs numbers refer to those matched with MitoCarta3.0. (**E**) Most enriched canonical pathways in the myotube transcriptome in the “completely reversed” clusters (e and f) that change with ammonium acetate (24hAmAc) treatment but return to baseline (UnT). (**F**) Most enriched canonical pathways in the myotube transcriptome in the “persistent” clusters (b and i) that change with 24hAmAc and do not improve with WD. All cellular experiments were done in *n* = 3 biological replicates. *P* value cutoff for all RNA-Seq DEGs was FDR < 0.05 by the Benjamini-Hochberg correction method. (**C**, **E**, and **F**) Green, decreased expression; red = increased expression. Significance cutoff for all pathways was –log(*P* value) ≥ 1.3 by the right-sided Fisher’s exact test.

Whole-cell transcript responses of both nuclear and mitochondrially encoded mRNA showed similar clustered responses during hyperammonemia and following ammonia withdrawal as in the proteome. The heatmap portrays these clusters while the Venn diagram identifies both shared and unique differentially expressed genes (DEGs) (Figure 4, A and B). Pathways relevant to mitochondrial oxidation and highly energy-dependent cellular regulatory functions (mRNA translation) were enriched in our data sets and included glycolysis, mitochondrial dysfunction, and NRF2-mediated oxidative stress response (Figure 4C). Interestingly, the cellular senescence pathway was also significantly enriched, suggesting a cellular adaptive response during mitochondrial oxidative dysfunction to maintain cell viability. To determine validated mitochondrial DEGs, we aligned the whole-cell transcriptome with MitoCarta3.0. We identified unique and shared DEGs between various data sets and clusters (Figure 4D). Unique pathways enriched in the whole-cell transcriptome cluster that had a “baseline return” with withdrawal included HIF1α, calcium, mTOR, and p70-S6K signaling pathways (Figure 4E). Unique pathways enriched in the whole-cell transcriptome cluster of DEGs that had “no change” with withdrawal included the tRNA charging pathway and EIF2 signaling (Figure 4F). Senescence-related, AMPK, PKA, and autophagy pathways were enriched in both transcriptome and proteome clusters, indicating there are some canonical DEGs in these pathways that responded to ammonia withdrawal and others that did not. Canonical pathways enriched in other clusters as well the MitoCarta3.0 verified mitochondrial DEGs are shown in Supplemental Table 4. We then determined if our supervised analyses were reproduced with unsupervised analyses of the data. Consistent with our supervised analyses, these unsupervised analyses showed hierarchical clustering of data from each treatment group. Furthermore, the untreated and withdrawal samples were more similar to each other than either was to the hyperammonemic data set (Supplemental Figure 3).

**Figure 5:**
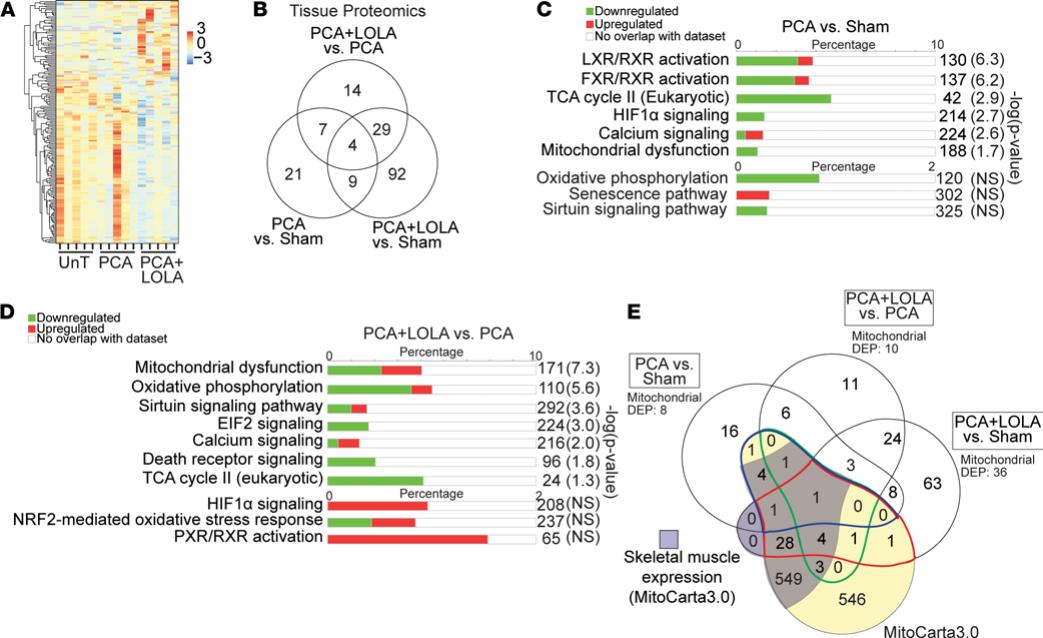
Skeletal muscle proteome from hyperammonemic or control rats with and without ammonia-lowering therapy. (**A**) Heatmap of the hierarchically arranged differentially expressed proteins (DEPs) of the proteome from gastrocnemius muscle from rats that underwent either a sham surgery (Sham) or a portacaval anastomosis (PCA) surgery with/without the ammonia-lowering agents L-ornithine-L-aspartate and rifaximin (LOLA). Each row represents 1 protein and each column represents 1 biological replicate (red = increased expression, blue = decreased expression). (**B**) Venn diagram of unique and shared DEPs in the tissue proteome from skeletal muscle from sham- and PCA-operated rats with/without LOLA. (**C**) Functional enrichment analyses of canonical pathways specifically curated for critical protein homeostasis and mitochondrial function in the skeletal muscle proteome from PCA rats treated with LOLA compared with Sham rats (green = decreased expression, red = increased expression). (**D**) Functional enrichment analyses of canonical pathways specifically curated for critical protein homeostasis and mitochondrial function in the skeletal muscle proteome from PCA rats treated with/without LOLA (green = decreased expression, red = increased expression). (**E**) Venn diagram of unique and shared DEPs in the muscle proteome data set overlaid with the genes listed in MitoCarta3.0 (shaded in yellow) and the DEPs known to be expressed in skeletal muscle within MitoCarta3.0 (shaded in purple). Verified mitochondrial DEPs in the PCA+LOLA versus Sham comparison (enclosed by a red line), PCA+LOLA versus PCA (green line), and PCA versus Sham (blue line). Mitochondrial DEPs refer to those matched with MitoCarta3.0. *P* value cutoff for all DEPs was set at *P* < 0.10 using a 2-tailed Student’s *t* test. All studies were performed in *n* = 5 biological replicates. Significance cutoff for all canonical pathways was set at –log(*P* value) ≥ 1.3 by the right-sided Fisher’s exact test.

Physiological relevance of our cellular data was established by quantifying the most upregulated and downregulated proteins in response to hyperammonemia and ammonia withdrawal in the gastrocnemius muscle from sham-operated, pair-fed control and hyperammonemic PCA rats and following ammonia-lowering therapy with L-ornithine-L-aspartate and rifaximin (LOLA). A heatmap and Venn diagram of the proteome in gastrocnemius from PCA rats showed significant differences from sham-operated controls and in response to LOLA (Figure 5, A and B). Manual curation of the most enriched mitochondrial regulatory pathways in the hyperammonemic PCA rats showed that components of oxidative phosphorylation, TCA cycle components, and bile salt signaling were decreased while calcium signaling, NRF2-mediated oxidative stress response, and HIF1 signaling pathways were increased compared with PCA rats treated with LOLA (Figure 5, C and D, and Supplemental Table 5). We then aligned the DEPs in the sham-operated control, PCA, and PCA treated with LOLA with MitoCarta3.0, which showed unique and shared DEPs with both mitochondrial and nonmitochondrial pathways (Figure 5E). Analyses of the mitochondrial and nonmitochondrial shared and unique DEPs that were involved in oxidative phosphorylation and protein homeostasis were similar to our observations in hyperammonemic myotubes.

### Hyperammonemia reversibly impairs responses to substrates, uncoupler, and inhibitors in muscle mitochondria in situ.

**Figure 6:**
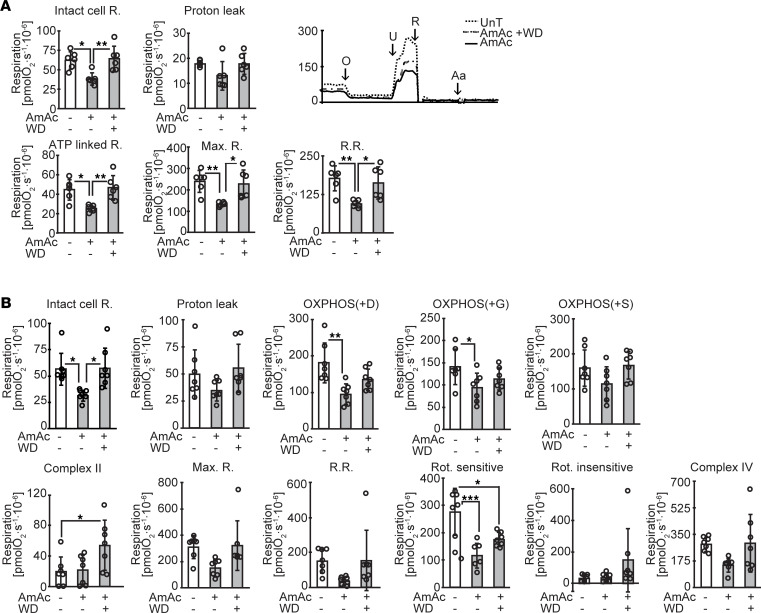
Hyperammonemia reversibly impairs mitochondrial respiration in differentiated C2C12 myotubes. (**A**) Representative oxygraph tracings from myotubes that were untreated (UnT), 24 hours ammonium acetate treated (AmAc), or following ammonia withdrawal for 24 hours (WD). Oxygen consumption was measured in intact nonpermeabilized myotubes in DMEM in response to ATP synthase inhibitor (oligomycin), uncoupler of oxidative phosphorylation trifluoromethoxy carbonylcyanide phenylhydrazone (FCCP) (U), rotenone, and a complex I inhibitor (R) and complex III inhibitor, antimycin A (Aa). (**B**) High-resolution respirometry to quantify oxygen consumption of permeabilized, differentiated C2C12 myotubes in mitochondrial respiration buffer by digitonin permeabilization and electron transport chain (ETC) complex–specific substrates and inhibitors sequentially in the concentrations as stated below. Proton leak, oxidative phosphorylation (OXPHOS) in response to malate (M), pyruvate (P), ADP (D), glutamate (G), and succinate (S), and maximum respiration (Max. R) and reserve respiratory capacity (R.R.) i.e., the response to FCCP (U), were quantified. Rotenone-sensitive and -insensitive respiration followed by complex IV function were measured. After initial stabilization, 2 mM malate and 2.5 mM pyruvate were added. This was followed by 4.1 μM digitonin to permeabilize the cell membrane without losing the integrity of cells or mitochondria for permitting entry of mitochondrial substrates inside the cells, 2.5 mM ADP, 10 mM glutamate, 10 mM succinate, 2 μM increments of FCCP for measuring maximum respiration, 375 nM rotenone (R), 125 nM Aa, 2 mM ascorbate (AT) and 2 mM tetramethyl phenylene diamine (TMPD) to test complex IV activity, and 50 mM sodium azide (Az) to inhibit complex IV activity. All data expressed as mean ± SD from at least 6 biological replicates for oxygraph studies in myotubes. *P < 0.05; **P < 0.01; ***P < 0.001 compared with respective controls by 1-way ANOVA followed by Bonferroni’s post hoc comparison tests.

**Figure 7:**
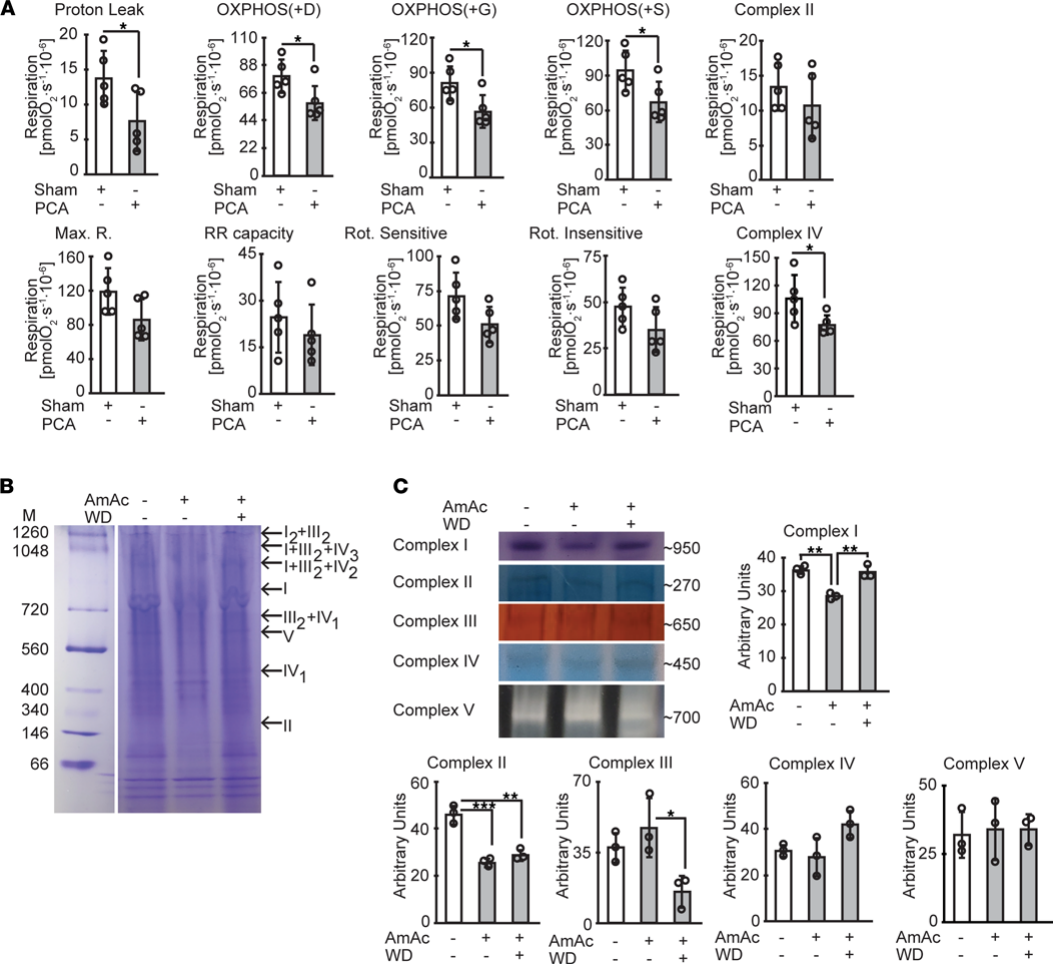
Hyperammonemia reversibly impairs mitochondrial respiration in gastrocnemius muscle tissue with disruption of supercomplex assembly in myotubes. (**A**) Mitochondrial oxygen consumption in permeabilized gastrocnemius muscle white fibers from hyperammonemic portacaval anastomosis (PCA) and sham-operated control rats in response to substrates and inhibitors of components of the electron transport chain (ETC) in the concentrations stated above. Saponin 50 μg/mL was used for permeabilization of fibers. (**B**) Blue native gel electrophoresis of isolated mitochondria to evaluate mitochondrial supercomplex assembly. (**C**) In-gel activity of mitochondrial complexes in UnT, AmAc-treated, and WD myotubes. All data expressed as mean ± SD from at least 6 biological replicates for oxygraph studies in myotubes, at least 3 biological replicates for other studies in myotubes, and 5 rats for each group *P < 0.05; **P < 0.01; ***P < 0.001 compared with respective controls by 1-way ANOVA followed by Bonferroni’s post hoc comparison tests or unpaired 2-tailed Student’s *t* test.

Mitochondrial oxidation serves to generate ATP synthesis from the coupling of electron transport across the ETC complexes to phosphorylation of ADP by complex V (ATP synthetase). Our unbiased data demonstrated reduction in specific components of the mitochondrial ETC during hyperammonemia and partial restoration of expression of these molecules following ammonia withdrawal/lowering. To dissect the mechanism of impaired mitochondrial oxygen consumption and to experimentally validate the unbiased data analyses, we performed the standard substrate, uncoupler, inhibitor titration (SUIT) protocol in intact and permeabilized myotubes treated with and without ammonia and following ammonia withdrawal (Figure 6, A and B) and in mitochondria isolated from myotubes treated with and without hyperammonemia (Supplemental Figure 4). Similar studies in permeabilized gastrocnemius muscle fibers from PCA and sham-operated rats were performed (Figure 7A). Oxygen consumption in response to complex I substrates (malate, pyruvate, and glutamate) was significantly lower in ammonia-treated myotubes compared with UnT myotubes and was reversed after 24 hours of ammonia withdrawal. Response to succinate, a substrate for complex II, also was lower in permeabilized, ammonia-treated myotubes and muscle from hyperammonemic PCA rats. Similar to previous observations in intact myotubes, maximum respiratory capacity measured in response to titration of the uncoupler FCCP was reduced in permeabilized, ammonia-treated myotubes. Response to the complex I inhibitor rotenone and complex III inhibitor antimycin A showed that rotenone-sensitive and rotenone-insensitive oxygen consumption were lower in ammonia-treated myotubes. Hyperammonemia also impaired complex IV function, measured as azide-sensitive oxidation of TMPD, an electron donor to cytochrome *c*, which in turn transfers the electron to oxygen to form water. These perturbations were consistent with our observations in myotubes, as we observed that gastrocnemius muscle from PCA rats had significantly impaired responses to substrates and uncoupler treatment compared with those from sham-operated pair-fed rats (Figure 7A). These impaired responses were reversed with ammonia-lowering therapy. Thus, our data in skeletal muscle mitochondria in situ show that hyperammonemia reversibly impairs the function of complexes I, II, and IV in myotubes and skeletal muscle in vivo.

In addition to the defects in substrate utilization by the ETC components, we also observed disruption of the supercomplexes on blue native polyacrylamide gel electrophoresis (BN-PAGE) during hyperammonemia with partial restoration with ammonia withdrawal (Figure 7B). Consistent with the substrate utilization studies and supercomplex assembly, in-gel activity of the ETC components showed partially reversible impairment during hyperammonemia in myotubes (Figure 7C). These experimental observations show that ammonia caused reversible mitochondrial dysfunction in specific components. We then evaluated the functional consequences of ammonia-induced mitochondrial perturbations in our cellular and in vivo models.

### Ammonia withdrawal reverses hyperammonemia-induced maladaptive mitochondrial oxidative responses.

**Figure 8:**
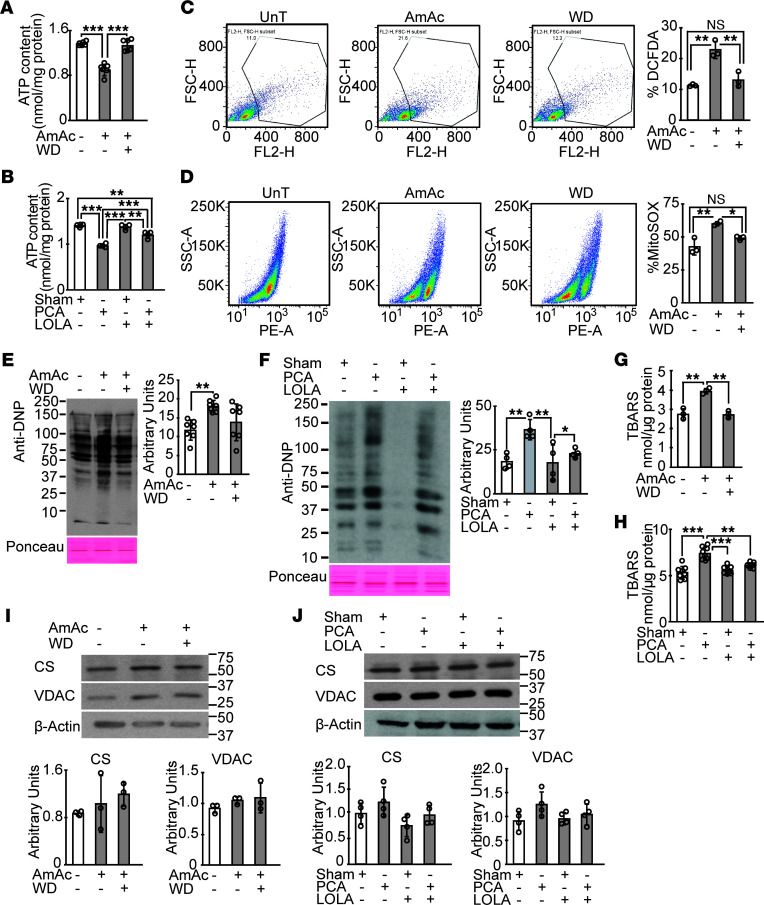
Reduction in ATP content and increased mitochondrial free radicals and oxidative modifications reversed by ammonia lowering. (**A**) Total ATP content in murine C2C12 myotubes treated with 10 mM ammonium acetate for 24 hours (AmAc) without or with withdrawal of ammonium acetate for 24 hours (WD) and untreated myotubes (UnT). (**B**) ATP content in gastrocnemius muscle from hyperammonemic portacaval anastomosis (PCA) or sham-operated control (Sham) rats treated with or without L-ornithine-L-aspartate and rifaximin (LOLA). (**C**) Flow cytometry–gated DCFDA fluorescence and percentage of DCFDA fluorescent cells in differentiated C2C12 myotubes treated with AmAc and WD. (**D**) Flow cytometry–derived fluorescence and percentage of cells stained by MitoSOX in response to AmAc and WD in differentiated myotubes. Representative immunoblots and densitometry for (**E** and **F**) carbonylated proteins and (**G** and **H**) lipid peroxidation quantified by thiobarbituric acid reactive substances (TBARS) in UnT, AmAc, and WD myotubes and in gastrocnemius muscle from PCA or Sham rats treated with or without LOLA. (**I**) Representative immunoblots and densitometry of citrate synthase (CS) and voltage dependent anion channel (VDAC) in UnT, AmAc, and WD myotubes. (**J**) Representative immunoblots and densitometry of CS and VDAC in gastrocnemius muscle from PCA or Sham rats treated with or without LOLA. All data expressed as mean ± SD from at least 3 biological replicates for experiments in myotubes and at least 5 rats in each group. *P < 0.05; **P < 0.01; ***P < 0.001 using 1-way ANOVA followed by Bonferroni’s post hoc comparison tests.

Consistent with our unbiased data and experimental ammonia-induced defects in ETC complexes and substrate responses, and previous reports of mitochondrial dysfunction during hyperammonemia (4, 18), total ATP content in the hyperammonemic myotubes was significantly lower than that in UnT or ammonia-withdrawn myotubes (Figure 8A). Physiological relevance was demonstrated by lower ATP content in gastrocnemius muscle from PCA rats compared with sham-operated control/PCA rats treated with LOLA (Figure 8B). In addition to the defects in mitochondrial oxidative function, our unbiased data showed changes in NRF2-mediated antioxidant responses. Consistently, generation of free radicals was significantly higher (*P* < 0.001) in ammonia-treated compared with UnT myotubes and those following ammonia withdrawal (Figure 8, C and D). We also determined that the activation of antioxidant responses was insufficient to mitigate the consequences of free radical generation as shown by higher expression of oxidative modifications including carbonylated proteins (Figure 8, E and F) and lipid peroxidation products (thiobarbituric acid reactive substances, TBARS) in hyperammonemic myotubes and muscle from rats compared with controls. Partial reduction in carbonylated proteins and TBARS following ammonia withdrawal/lowering in hyperammonemic myotubes and gastrocnemius muscle from PCA rats (Figure 8, G and H) show that the adaptive antioxidant response to hyperammonemia was not sufficient to overcome mitochondrial ROS in skeletal muscle. Mitochondrial oxidative dysfunction during hyperammonemia was not accompanied by changes in mitochondrial content (citrate synthase, and outer membrane protein and voltage dependent anion channel [VDAC] expression) in myotubes (Figure 8I) or rat skeletal muscle (Figure 8J).

### Hyperammonemia causes cataplerosis that is reversed by lowering ammonia and reversible senescence in myotubes and skeletal muscle.

**Figure 9:**
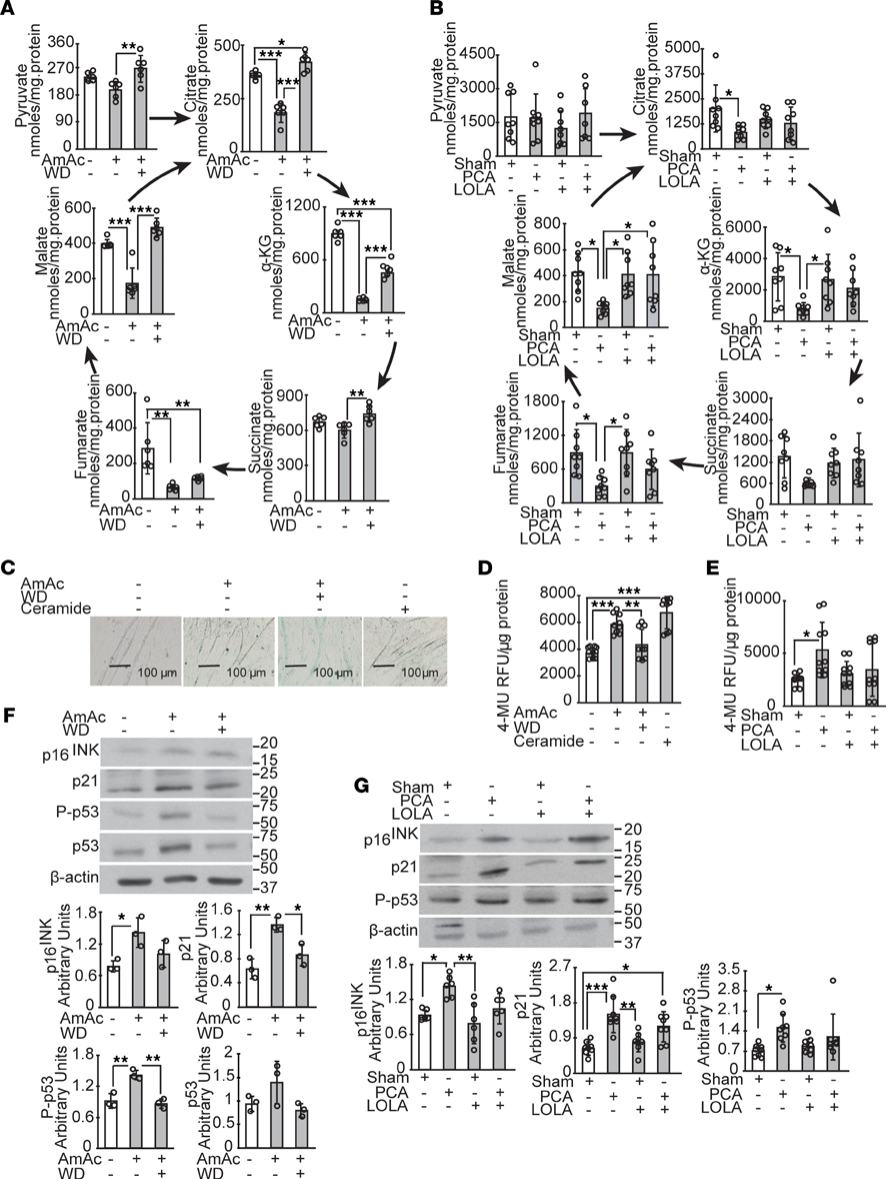
Reduced intermediary metabolites and postmitotic senescence reversed by ammonia lowering. (**A**) Pyruvate and TCA cycle intermediates in differentiated myotubes that were treated with 10 mM ammonium acetate for 24 hours (AmAc), following withdrawal of ammonium acetate for 24 hours (WD), and untreated (UnT) myotubes. (**B**) Pyruvate and TCA cycle intermediates in gastrocnemius muscle from hyperammonemic portacaval anastomosis (PCA) or sham-operated control (Sham) rats treated with or without L-ornithine-L-aspartate and rifaximin (LOLA). (**C**) Representative photomicrographs of differentiated C2C12 myotubes stained for senescence-associated β-galactosidase activity. Myotubes incubated with C2 ceramide (50 μM) for 8 hours were used as a positive control. (**D** and **E**) Senescence-associated β-galactosidase activity was quantified and expressed as 4-methyl umbelliferone (4-MU) fluorescence normalized to protein content in UnT, AmAc, and WD myotubes and skeletal muscle tissue from PCA and sham rats treated with and without LOLA. (**F** and **G**) Representative immunoblots and densitometry of senescence marker proteins, p16, p21, and phosphorylated p53 in myotubes and gastrocnemius muscle of PCA and sham rats treated with/without LOLA. All data expressed as mean ± SD from at least 3 biological replicates for experiments in myotubes and at least 5 rats in each group. *P < 0.05; **P < 0.01; ***P < 001 using 1-way ANOVA followed by Bonferroni’s post hoc comparison tests.

Our proteomics data showed perturbations in expression of enzymes regulating the TCA cycle intermediary metabolites in response to ammonia treatment in myotubes. Previous studies have shown cataplerosis of critical TCA cycle intermediate αKG during hyperammonemia (4). To determine if ammonia lowering restored intermediary metabolite levels, we quantified the cellular and tissue concentrations of pyruvate, an oxidative substrate for the TCA cycle, as well as the intermediates in the TCA cycle. Cellular concentrations of pyruvate and all TCA cycle intermediates except succinate were significantly lower in ammonia-treated compared with UnT myotubes, and these perturbations were reversed by ammonia lowering for 24 hours (Figure 9A). Similar to the observations in ammonia-treated myotubes, skeletal muscle from PCA rats had lower concentrations of TCA cycle intermediates compared with those in pair-fed, sham-operated rats, with no significant response to ammonia lowering of most metabolites (Figure 9B).

In addition to lower ATP levels and free radical generation, mitochondrial dysfunction has been identified to initiate cellular senescence (19). Unlike that in replicating cells, senescence in postmitotic cells is manifested by a senescence-associated phenotype of reduced cell size, as well as functional responses that include lower protein synthesis, mitochondrial dysfunction, and increased expression of β-galactosidase and cell cycle regulatory molecules p16^INK^ and p21 (15, 16, 20). In the present studies, we identified enrichment of senescence pathways in our unbiased data analyses. We also observed increased β-galactosidase in hyperammonemic myotubes and skeletal muscle with the novel observation of reduction in expression after ammonia withdrawal (Figure 9, C–E). Consistently, canonical proteins involved in senescence, including p16^INK^, p21, and phosphorylated p53, had increased expression with ammonia treatment that was reversed with ammonia withdrawal in myotubes and lowering in vivo (Figure 9, F and G). Even though the cell cycle regulatory molecules are implicated in replicative senescence, our data suggest a role of these molecules in nonmitotic cells also. These data suggest that ammonia lowering may be a potential approach for restoring functional responses to anabolic stimuli during senescence.

### Hyperammonemia sensitizes myotube responses to rechallenge.

To determine if ammonia lowering alters responses to subsequent hyperammonemia, mitochondrial oxidative function and senescence-associated molecular phenotype were determined in hyperammonemic myotubes that were ammonia treatment naive and those rechallenged with ammonia following initial hyperammonemia and ammonia lowering. Reduction in myotube diameter was less during rechallenge than during hyperammonemia in naive cells (Supplemental Figure 5A). Mitochondrial oxidative function was significantly lower with hyperammonemia in rechallenged compared with naive cells (Supplemental Figure 5B). However, ATP content and carbonylated proteins were not different between hyperammonemia in naive and rechallenged cells (Supplemental Figure 5, C and D). Interestingly, senescence marker proteins p16^INK^ and p21 were significantly lower in myotubes rechallenged with ammonia than hyperammonemia in naive cells (Supplemental Figure 5E). These data suggest differential sensitization of molecules/pathways to ammonia rechallenge.

## Discussion

Ammonia, a cytotoxic cellular metabolite and interorgan metabolic exchange molecule, is metabolized by mitochondria to urea in hepatocytes and other molecules in nonhepatic tissue (2). In the present studies we dissected the mechanistic basis of the consequences of nonureagenic ammonia disposal using complementary comprehensive bioinformatics analyses with experimental validation of critical observations. We observed that skeletal muscle ammonia disposal causes mitochondrial oxidative dysfunction and activation of components of cellular senescence. Mitochondrial and cellular plasticity was demonstrated by partial reversal of these responses by ammonia lowering. Global responses included changes in protein and/or RNA expression during hyperammonemia and following ammonia withdrawal. We organized these changes into different clusters in a supervised manner based on analyses of specific perturbations, including reversibility of these alterations. Consistently, unsupervised analyses to determine the sample concordance yielded clusters that can be used in future studies to identify a novel hyperammonemic or withdrawal “signature.” Substrate oxidation by different ETC complexes as well as cellular and tissue concentrations of ATP and TCA cycle intermediates were decreased during hyperammonemia and were reversed by ammonia lowering. Consistent with maintained cell viability despite persistence of mitochondrial dysfunction, an increase in senescence markers was observed. Finally, ammonia withdrawal/lowering restored mitochondrial oxidative function and reversed the ammonia-induced increased expression of senescence markers. The translational relevance of our bioinformatics analyses and cellular experiments were established by the use of ammonia-lowering interventions in vivo.

A comprehensive array of systems biology–based, unbiased approaches including supervised and unsupervised analyses showed global perturbations in mitochondrial oxidative function, including those in all components of the ETC, with enrichment in antioxidant responses and senescence components, including the sirtuin deacetylases. Sirtuins are deacetylases that are dependent on cellular NAD^+^/NADH ratio, and sirtuin expression/activity suppresses senescence via a number of mechanisms. Impaired mitochondrial oxidative phosphorylation is consistent with lower NAD^+^/NADH during hyperammonemia reported by us earlier (4) and can explain the changes in the sirtuin pathway during hyperammonemia. Ammonia-induced mitochondrial dysfunction has also been reported in the brain, which suggests that these perturbations in multiple pathways may occur in organs beyond the skeletal muscle (4, 18). Even though mitochondrial oxidative response is decreased with muscle hyperammonemia (4), the specific defects in various ETC complexes or their components have not been reported to our knowledge. In the present studies, in our unbiased data analyses, we identified that the expression of a number of (but not all) components of each ETC complex is decreased. The majority of decreases were noted in complexes I, III, and IV, especially in those components that accept electrons rather than transfer electrons to downstream complexes/components or the matrix. These suggest a greater susceptibility of subcomponents that are more electronegative than downstream components and need to be evaluated further. Interestingly, recovery following ammonia withdrawal was noted primarily in complexes I and IV, but recovery was greater in the portions involved in electron transfer to downstream components or electron transfer to the matrix. Expression of the DEPs that changed with ammonia in the oxidative phosphorylation pathway was not completely reversed and explains the partial recovery following ammonia withdrawal. Our data show that not all perturbations were reversed during ammonia withdrawal in myotubes or ammonia lowering in vivo in PCA rats. Differential sensitivity of molecules/pathways that are irreversibly altered during hyperammonemia may be due to translational/posttranslational modifications. Global translation defects and increased protein nitration have been reported during hyperammonemia (21, 22), but whether such alterations are responsible for partial or nonrecovery after ammonia withdrawal requires future studies. Another potential reason for failure to respond may be differences in the turnover rates of individual molecules/regulatory proteins during hyperammonemia or nutrient supply, including those promoting anaplerosis following ammonia withdrawal, and therefore, longer periods of ammonia lowering with replenishment of nutrients may promote recovery of molecules/pathways that did not show a response with the current experimental approach. Alterations in expression of ammonia transporter during hyperammonemia and the time for such alterations to be restored to baseline activity can also contribute to the temporal clustering of differential responses. Our interesting observations that some of the pathways/molecules continue to change in the same direction as occurred during hyperammonemia (progressive cluster) or only recovered partially after ammonia withdrawal or lowering (persistent cluster) contribute to the heterogeneity of responses to ammonia withdrawal. These observations have high translational relevance because the recovery for different adverse consequences of hyperammonemia may need tailored interventions beyond only ammonia lowering that include providing substrates for metabolites/protein synthesis that accelerate recovery (e.g., anaplerotic agents, sirtuin activators, essential amino acids, antioxidants).

Consistent with our unbiased data analyses, our experimental studies to dissect the specific ETC component defects showed that responses to substrates for complexes I, III, and IV were decreased during hyperammonemia and partially recovered with withdrawal. Each ETC component is critical in the oxidation of its upstream component, a process that culminates in ATP synthesis. Our in-gel activity assays were consistent with the impaired oxidative function observed and showed that in our model, in vitro activity correlated with functional responses. Others, however, have noted that the expression (of ETC components) in in vitro activity and functional assays measuring oxygen consumption do not yield consistent correlations, which may be related to a number of factors, including the formation of supercomplexes in situ on the inner mitochondrial membrane (23, 24). Disruption of the supercomplexes observed in our studies during hyperammonemia could explain the consistency in our responses. Even though we did not study the mechanisms of supercomplex disruption, others have reported that free radical generation can alter the inner mitochondrial membrane characteristics with loss of the assembly of the various components of the ETC (24, 25). Our observation of increased lipid peroxidation products suggests that an alteration in mitochondrial membrane lipids may be a potential contributor to the loss of supercomplex assembly. Another potential mechanism for disassembly of the supercomplexes is a change in the protein structure that may occur as part of the oxidative modification of proteins as noted in our data as well as those reported by others (26) during hyperammonemia. Potential mechanisms of restoration (even if partial) of both ETC responses to substrates and supercomplex assembly by ammonia withdrawal/lowering include reversibility of ammonia-induced protein or inner mitochondrial membrane modification, antioxidant responses that can restore protein function, a rapid translational upregulation of critical components of the ETC, or varying contributions from each of these factors.

Our metabolic studies show that ammonia-induced cataplerosis was reversed by ammonia withdrawal/lowering both in myotubes and in muscle tissue from hyperammonemic rats. These data are consistent with previous reports that ammonia causes cataplerosis of αKG (4) and extend those observations by demonstrating that cellular adaptive mechanisms can restore intermediary metabolites upon ammonia withdrawal (4). Anaplerotic substrates in the skeletal muscle include branched chain amino acids and glucose (5). Since no additional anaplerotic substrates were provided, restoration of mitochondrial intermediary metabolites, even if partial, with ammonia lowering or withdrawal would require endogenous generation or intake from the culture medium in myotubes. We and others have previously shown increased autophagy during hyperammonemia (27) that can explain the reduced but maintained mitochondrial oxidative function and the restoration of intermediary metabolites with ammonia withdrawal.

Another interesting observation is the ammonia-induced development of senescence in skeletal muscle. Such a response is similar to the stress-induced premature senescence reported with sublethal doses of hydrogen peroxide and other chemicals (28). The dose and duration of hyperammonemia in our studies induce a hyperammonemic stress that shares a number of characteristics with the integrated stress response (3, 29). Despite continued stress, the mechanism of maintained cell viability could be due to the development of senescence. The present studies show increased senescence-associated β-galactosidase activity in myotubes, consistent with previously reported phenotypic and functional consequences of senescence during hyperammonemia, including a loss of muscle mass and myotube diameter, reduction in protein synthesis, and decreased response to anabolic stimuli (29). Recently, mitochondrial dysfunction–associated senescence has been reported and is characterized by a distinct secretory phenotype (19), but the mechanisms of mitochondrial dysfunction as a causal or an epiphenomenon in initiating such responses is not yet established. Our unbiased and experimental studies demonstrate the direct causal nature of an ammonia-induced senescence phenotype with known canonical senescence protein expression that is partially reversed with ammonia withdrawal. Such an observation is consistent with data that cellular senescence is potentially reversible (30). The mechanisms by which increased canonical cell cycle regulatory proteins induce senescence in postmitotic cells need to be dissected, but the regulation of mRNA translation by cyclin-dependent kinases is a potential regulatory mechanism (31, 32). Given the role of ammonia in astrocyte senescence (12) in humans and in rice leaves in plants (33) and emerging evidence of the role of ammonia in Alzheimer’s disease (34) and the current data in skeletal muscle, targeting ammoniagenesis or enhancing ammonia removal are potential areas of therapeutic development to reverse senescence in postmitotic cells.

Our likely highly novel data show that ammonia rechallenge worsens mitochondrial oxidative function compared with the responses during hyperammonemia. However, these perturbations in mitochondrial function are not immediately translated into functional responses as measured by ATP content or carbonylated proteins. This may be due to the differential temporal course of responses during recurrence of hyperammonemia, and this course is similar to the variable responses of different pathways/molecules during ammonia withdrawal. Interestingly, myotube diameter and senescence markers p16^INK^ and p21 were affected less during ammonia rechallenge than with hyperammonemia in naive cells. Whether these are due to differences in regulatory responses or desensitization of certain responses to recurrence of hyperammonemia needs to be evaluated in future studies including responses to recurrent hyperammonemia in vivo following ammonia-lowering therapies. The impact of removal of the medium and replacement with fresh medium on the rechallenge responses needs to be considered during interpretation of these data. Notwithstanding these confounders, these potentially novel data lay the foundation for human studies on recurrence of sarcopenia and hepatic encephalopathy, both of which are known consequences of hyperammonemia after discontinuation of ammonia-lowering therapies.

Previous reports have suggested that prior ethanol exposure sensitizes the skeletal muscle to ammonia via increased expression of regulated ammonia transporter, RhBG (35). Our current studies show that ammonia rechallenge also sensitizes myotubes to some of the effects of ammonia, specifically mitochondrial oxidative function, but desensitizes other effects, including senescence markers with no significant difference in ATP content or protein carbonylation between ammonia treatment and rechallenge. The differential effects may be due to the variable sensitivity of different pathways as noted in our unbiased data analyses during ammonia withdrawal or altered ammonia transport/metabolism during the rechallenge. The mechanism for failure of ammonia rechallenge to reinitiate senescence markers is currently not known but may be related to persistence of hyperammonemia-induced adaptive responses. In vivo studies are needed to determine the physiological and translational relevance of variable in vitro responses to ammonia rechallenge because recurrence of hyperammonemia can occur in human disease after discontinuation of ammonia-lowering therapy.

In summary, our observations using a complementary approach of supervised and unsupervised unbiased data analyses with experimental validation show that hyperammonemia causes a distinct, partially reversible mitochondrial oxidative dysfunction, oxidative stress response, and cellular senescence in postmitotic muscle cells and tissue. Expression of mitochondrial and nuclear encoded components of ETC complexes I, III, and IV was reversibly decreased and was associated with oxidative dysfunction and free radical generation. Hyperammonemia also induced a senescence phenotype and functional alterations that were partially reversed by lowering ammonia. These data in conjunction with clinical use of ammonia-lowering measures provide a novel approach to reverse senescence in skeletal muscle and potentially other postmitotic cells.

## Methods

All reagents were obtained from MilliporeSigma, and all antibodies were obtained from Cell Signaling Technology, unless specifically stated. The details of all key reagents are shown in Supplemental Table 6.

### In vitro studies in myotubes

Murine C2C12 myoblasts (ATCC) were grown to confluence in proliferation medium (DMEM with 10% fetal bovine serum), followed by differentiation in DMEM with 2% horse serum for 48 hours, and exposed to 10 mM ammonium acetate, a concentration that reproduces the concentrations in skeletal muscle in cirrhosis as previously reported by us (36). Responses to ammonia withdrawal were studied in myotubes treated with ammonium acetate for 24 hours followed by media removal and replacement with fresh differentiation medium. Myotube diameter was measured as previously described by us, and data were expressed as percentage of UnT controls (36).

### Mitochondrial isolation

Mitochondria were isolated from differentiated C2C12 myotubes as described earlier (37). In brief, washed myotubes were lysed and centrifuged, the cell suspension was homogenized, and mitochondria were isolated by centrifugation for 20 minutes at 10,000*g* at 4°C. Mitochondrial protein was quantified and purity established by immunoblots for proteins unique to mitochondria, nuclei, and endoplasmic reticulum.

### In vivo studies

Postpubertal male Sprague-Dawley rats aged 9 weeks, with either end-to-side PCA or sham surgery, were obtained from Charles River Inc., where animals were housed individually in a 12-hour light/12-hour dark cycle as described earlier (10). PCA rats were fed ad libitum while the sham-operated rats were pair fed with the PCA rats as described earlier (10). Rifaximin and LOLA were administered to lower blood ammonia in rats as described previously (10). Gastrocnemius muscle was harvested, a part of which was used for mitochondrial oxygen consumption studies, and the rest was frozen for proteomics, metabolite, and chemical assays.

### Unbiased approaches to determine alterations in hyperammonemic myotubes

#### Whole-cell transcriptomics.

To gain insights into global alterations in mitochondrial metabolic and adaptive pathways, including senescence responses to hyperammonemia and ammonia withdrawal, whole-cell RNA-Seq from differentiated C2C12 myotubes that were UnT or treated with 10 mM ammonium acetate was performed as described previously (21). In brief, total RNA was extracted, quality was determined with an Agilent 2100 bioanalyzer, RNA-Seq libraries were generated and sequenced, and bioinformatics analyses were done by Novogene using TopHat2 as a mapping tool. Gene expression was quantified by HTseq v0.6.1 to count the number of reads mapped to each gene, and read counts were normalized by conversion to fragments per kilobase of transcript per million mapped reads. DESeq2 (1.18.0) was used for differential gene expression analyses.

#### Proteomics studies.

Untargeted proteomics analyses were performed in whole cells and mitochondria isolated from UnT myotubes, in those treated with 10 mM ammonium acetate, and after ammonia withdrawal using methods previously described by us (38). Physiological relevance was established by untargeted proteomics in gastrocnemius muscle from the hyperammonemic PCA and sham-operated rats with/without LOLA to lower ammonia using methods previously reported by us (10, 38). In brief, samples were homogenized by sonication, centrifuged, and digested with trypsin. Samples were then reduced by dithiothreitol, alkylated by iodoacetamide, desalted, lyophilized, and reconstituted in acetic acid for liquid chromatography mass spectrometry analysis.

Digested peptides were analyzed on a Thermo Fisher Scientific UltiMate 3000 UHPLC system interfaced with an Orbitrap Fusion Lumos Tribrid mass spectrometer (Thermo Fisher Scientific) as previously described (38). In brief, the digest was analyzed using the data-dependent multitask capability of the instrument, acquiring full scan mass spectra using a Fourier Transform Orbitrap analyzer to determine peptide molecular weights and collision-induced dissociation tandem mass spectrometry (MS/MS) product ion spectra with an ion trap analyzer to determine the amino acid sequence in successive instrument scans. Data were analyzed using the Proteome Discoverer V2.3 (Thermo Fisher Scientific) with the search engine Sequest-HT integrated in the Proteome Discoverer software. For the cell and mitochondrial samples, the Uniprot mouse protein sequence database containing 16,996 entries (downloaded on July 9, 2019) with an automatically generated decoy database (reversed sequences) was used to search the MS/MS spectra. For the rat muscle samples, the Uniprot rat protein database containing 35,859 entries (downloaded on July 15, 2021) with an automatically generated decoy database (reversed sequences) was used to search the MS/MS spectra. An FDR was set to 1% for both peptide and protein identification and calculated using the number of identified peptides/proteins from the decoy database divided by the total number of identified peptides/proteins. Two peptides were required for positive protein identification to decrease the chance of false discovery by a random match. A precursor intensity-based, label-free quantification was achieved by the Minora Feature Detector node of Proteome Discoverer using peptide precursors in MS full scans and normalized by total amount of peptide in each sample. The peptides used in this quantitation correspond to unique + razor. At least 2 quantifiable peptides are required for a protein. Details of these methods have been described in detail earlier (38).

#### Unbiased data analysis.

Log_2_ expression ratios were calculated for each of the unbiased treatment comparisons (i.e., myotubes treated with 24 hours of ammonium acetate vs. UnT myotubes, myotubes treated with 24 hours of ammonium acetate vs. those following ammonia withdrawal, and myotubes following ammonia withdrawal vs. UnT myotubes) for RNA-Seq and proteomics to determine differential expression. Supervised analyses of temporal clusters of DEPs or DEGs were analyzed as previously reported (38) and shown in Figure 1A and Supplemental Table 7. These clusters included molecules with differential expression that either increased or decreased (compared with UnT cells) with 24-hour ammonium acetate treatment, and with ammonium acetate withdrawal, the expression increased (or decreased) further (compared with UnT cells) in the same direction (i.e., clusters a and j, “progressive”), remained at the same differential expression as with ammonia treatment (i.e., clusters b and i, “persistent”), partially returned toward UnT levels (i.e., clusters d and g, “partially reversed”), completely returned to UnT levels (i.e., clusters e and f, “completely reversed”), or increased (or decreased) beyond UnT levels in the opposite direction (i.e., clusters c and h, “overcorrection”). Venn diagrams were constructed and functional enrichment analysis of pathways was performed using these clusters as previously reported (38). We then performed artificial intelligence–based, unsupervised analysis using hierarchical complete-linkage clustering of features using a parameter of 10 k-means clusters to generate a comparable number of clusters as in the supervised analysis and hierarchical complete-linkage clustering of samples allowing for identification of samples with the greatest concordance. Transcriptomic and proteomic data from whole cells, mitochondria, and muscle tissue were thus evaluated using supervised and unsupervised approaches.

Functional analyses were generated with the log-transformed expression ratios through the use of IPA (QIAGEN, https://www.qiagenbio- informatics.com/products/ingenuity-pathway-analysis), and heatmap and canonical pathway overlays were generated as previously described (21, 38). All Venn diagrams and heatmaps were created with R (v4.0.2). Venn diagrams were created using the venn (https://CRAN.R-project.org/package=venn), ggplot2 (https://ggplot2.tidyverse.org), and ggpolypath (https://cran.r-project.org/package=ggpolypath) packages to identify unique and common genes in different treatment groups. Heatmaps were created using the pheatmap (https://cran.r-project.org/package=pheatmap), DESeq2, gplots (https://CRAN.R-project.org/package=gplots), and dendextend packages (39) to identify patterns of gene expression by sample across treatment groups. Functional enrichment analyses were performed with IPA. The significance cutoff for proteins in the proteomics analysis was taken at *P* < 0.05 (2-tailed Student’s *t* test). The significance cutoff for genes in the RNA-Seq analyses was taken at an FDR less than 0.05 (Benjamini Hochberg adjustment). The significance cutoff for canonical pathways was set at –log(*P* value) > 1.3 (right-tailed Fisher’s exact test). Skeletal muscle pathways were derived from the IPA knowledge database and manually curated to ensure known expression in skeletal muscle.

The complete list of C2C12 whole-cell proteins and C2C12 mitochondrial proteins detected by unbiased proteomics using liquid chromatography/MS/MS were compared with the proteins from the MitoCarta3.0 database (Broad Institute, https://www.broadinstitute.org/mitocarta/mitocarta30-inventory-mammalian-mitochondrial-proteins-and-pathways) (17). Genes identified in MitoCarta3.0 as present in skeletal muscle tissue were also compared against our data in Venn diagram format to identify unique and common genes or proteins. Within these comparisons, 7 sets of proteins were identified and quantified as shown in Supplemental Tables 8 and 9 based on whether they were present in 1 or more of the data sets: whole-cell proteomics, mitochondrial proteomics, and MitoCarta3.0.

For canonical senescence genes, genes/proteins from our studies were compared with the CSGene (40) and CellAge (41) databases. We used both these databases that have shared and unique genes due to differences in the approach to create the databases. The unique genes that were not shared with those in the CSGene and CellAge databases provide potential genes that need to be evaluated for their relevance in skeletal muscle senescence in future studies. DEGs and DEPs from all data sets described heretofore as well as their overlap with MitoCarta3.0 and senescence databases are shown in Supplemental Table 10.

### Targeted metabolomics

Metabolic intermediates in the TCA cycle were quantified in UnT, ammonia-treated, and after ammonia withdrawal myotubes and gastrocnemius muscle from PCA and sham-operated rats using methods previously reported (42).

### Experimental validation of unbiased data analyses focusing on mitochondrial dysfunction and cellular senescence during hyperammonemia

#### Mitochondrial oxidative function in intact and permeabilized myotubes.

Mitochondrial function in intact myotubes and in permeabilized myotubes using SUIT protocols was measured with high-sensitivity respirofluorometry as described previously (37, 43). In brief, differentiated, intact myotubes in the UnT, hyperammonemia, and ammonia withdrawal groups were suspended in differentiation medium and treated with oligomycin to determine ATP synthesis from phosphorylation linked to oxidation. Maximum respiratory capacity was quantified using the protonophore and uncoupler of oxidative phosphorylation, FCCP. Responses to rotenone, a complex I inhibitor, and antimycin A to measure nonmitochondrial respiration were quantified as described earlier (37, 43). To determine responses to substrates for the ETC complexes, studies were performed in permeabilized myotubes as reported earlier (37, 43). In brief, following measurement of oxygen consumption in intact differentiated C2C12 myotubes in mitochondrial respiration medium, MiR05, digitonin was used to permeabilize cells. Oxidation coupled to phosphorylation was quantified in response to substrates for complexes I and II and ADP. Mitochondrial substrates malate, pyruvate, and glutamate were added to measure complex I function. Succinate was then added as a complex II substrate. Maximal oxidative capacity, or maximum respiration, was measured by the addition of FCCP, followed by rotenone to inhibit electron flow across complex I. Antimycin A (complex III inhibitor) was then added to determine nonmitochondrial residual oxygen consumption rate. Uncoupled complex IV oxidation rate was calculated using sodium azide followed by TMPD and ascorbate.

Oxygen concentration and flow rates were recorded at 2-second intervals to measure oxygen consumption rates using DatLab2 from Oroboros. Data were generated from at least 6–7 biological replicates after calibration of the oxygen sensors and instrument background corrections. All data were expressed as oxygen consumption in pmol/s normalized to cell number or wet weight of tissue to allow for comparisons across experiments.

#### Oxygen consumption in isolated mitochondria.

Mitochondria were isolated from murine myotubes treated with/without 10 mM ammonium acetate as above. Equal quantities of mitochondria measured by protein content were added to each chamber of the respirofluorometer, and responses to substrates, inhibitors, and uncoupler as in permeabilized cells were quantified.

#### Mitochondrial respiration in permeabilized muscle tissue.

Studies were performed in carefully weighed, fresh gastrocnemius muscle from 5 rats in each group. Muscle tissue was gently dissected and fibers separated, followed by permeabilization using saponin as described previously (37, 43). Oxygen consumption at 37°C was measured using a high-sensitivity respirofluorometer as described earlier (Oroboros) (43, 44).

#### Immunoblots.

Immunoblots for protein extracts were performed to quantify mitochondrial proteins VDAC and citrate synthase as measures of mitochondrial mass using protocols described by us earlier (4, 43). In brief, electrophoresis of 30 μg total protein was performed on a 4%–20% tris-glycine gel. The protein was electrotransferred to PVDF membrane and incubated in primary followed by secondary antibody (Supplemental Table 6), and blots were developed followed by densitometry using ImageJ (NIH) to quantify the blots.

#### ATP measurement.

Total cellular ATP content in the gastrocnemius muscle from gastrocnemius skeletal muscle and UnT, ammonia-treated, or ammonia-withdrawn C2C12 myotubes was quantified using a bioluminescence assay with a commercial kit per manufacturer instructions (Molecular Probes) as previously described (4, 43).

#### Protein carbonylation.

Oxidative stress–induced posttranslational modification of proteins was determined by derivatization of the carbonyl group using 2,4 dinitrophenyl and detected by immunoblots for protein carbonylation and as described by us previously (4, 43).

#### Mitochondrial supercomplex assembly.

Components of the ETC on the inner mitochondrial membrane exist as supercomplexes on the inner mitochondrial membrane, and disruption of these supercomplexes results in dysfunction (24, 45). We evaluated if supercomplexes were disrupted during hyperammonemia in BN-PAGE using methods reported previously (37, 45).

#### Free radical generation.

The cell-permeable fluorescent dye chloromethyl, 2,7, dichlorodihydro fluorescein diacetate or mitochondrial specific probe MitoSOX was used to quantify free radical generation using flow cytometry as described by us previously (43).

#### Mitochondrial ETC complex function using in-gel activity assay.

In-gel activity of individual ETC complexes was performed as reported by others with minor modifications (45). In brief, a high-resolution BN-PAGE was performed in mitochondria isolated as described above from UnT or hyperammonemic myotubes. Protein was quantified by a bicinchoninic acid assay, and mitochondria were lysed by incubation with 8 μg digitonin/μg protein for 20 minutes on ice. Proteins were separated by electrophoresis in a 4%–12% Bis-Tris native gel. Gels were then incubated with the specific substrate for each of the ETC complexes. For complex I activity, gel was stained with a solution containing 2 mM Tris-HCl pH 7.4 with 0.1 mg/mL of NADH and 2.5 mg/mL nitro blue tetrazolium chloride (NBT). Complex II activity was measured by staining the native gel with 10 mL substrate solution (20 mM of sodium succinate, 25 mg NBT 0.2 mM of 250 mM phenazine methosulphate in 5 mM Tris-HCl buffer). Complex III and IV activities were measured by staining the gel with solution containing 10 mL of substrate with 5 mg diaminobenzidine, 10 mg cytochrome *c*, and 9 mL of 50 mM phosphate buffer pH 7.4. For Complex V activity, the gel was incubated in the substrate solution containing 35 mM Tris, 270 mM glycine,14 mM MgSO_4_, 10 mM ATP, and 0.2% Pb(NO_3_)_2_ in 10 mL of water. The reaction was stopped by washing the gels with deionized water and treatment with 10% acetic acid.

#### Quantitative assay for β-galactosidase.

A fluorometric assay was used to quantify senescence-associated β-galactosidase activity in myotubes and muscle tissue using a modification of methods previously described (46). In brief, cells or about 1 mg of frozen tissue were treated with lysis buffer consisting of 5 mM 3(3-cholamidopropyl) dimethylammonio)-1-propane sulfate, 40 mM citric acid, 40 mM sodium phosphate, 0.5 mM benzamide, 0.25 phenylmethylsulfonyl fluoride, with the lysate vortexed vigorously and centrifuged at 12,000*g* for 5 minutes at 4°C. The supernatant was mixed with an equal volume (150 μL) of 2× reaction buffer (40 mM citric acid, 40 mM sodium phosphate, 300 mM sodium chloride, 10 mM β-mercaptoethanol, 4 mM magnesium chloride, 1.7 mM 4-methylumbelliferyl-d-galactopyranoside in water). A total of 50 μL of this reaction mixture was then added to 500 μL of 400 mM sodium bicarbonate stop solution. All reactions were carried out at 4°C. Fluorescence readings were obtained with an excitation at 360 nm and emission at 465 nm on a PerkinElmer plate reader (model 1420-050) and data expressed as RFU/μg protein.

#### β-Galactosidase staining of myotubes.

Staining for β-galactosidase was performed using modifications of a protocol previously published by others (47). In brief, after myotubes grown on a coverslip were washed, they were washed in PBS, were fixed for 5 minutes with 3% formaldehyde, were washed and incubated at 37°C with fresh X-Gal (Thermo Fisher Scientific) working solution (1:40 dilution in X-gal dilution buffer: potassium ferricyanide crystalline 5 mM, potassium ferricyanide trihydrate 5 mM, magnesium chloride 2 mM in PBS protected from light) for 12 hours, and were rinsed with PBS for 5 minutes and then briefly with distilled water. Nuclear fast red was used for counterstaining for 5 minutes, and myotubes were mounted with aqueous mounting medium.

#### Rechallenge experiments.

To determine if responses to ammonia exposure in naive cells were different from those during rechallenge with ammonia, differentiated myotubes were treated with 10 mM ammonium acetate, followed by ammonia withdrawal for 24 hours by replacement with fresh DMEM with 2% horse serum, and treated with ammonium acetate for another 24 hours. Myotube diameter, mitochondria oxidative function in intact myotubes, ATP content, expression of carbonylated proteins, and expression of senescence markers p16^INK^ and p21 were compared in hyperammonemia in naive cells and in response to ammonia rechallenge.

#### Data accessibility.

All unbiased data have been uploaded to publicly accessible repositories. The RNA-Seq data have been deposited to GitHub at https://github.com/dasaraslab/Unbiased (commit ID 8d1a104). The mass spectrometry proteomics data have been deposited to the ProteomeXchange Consortium via the PRIDE (48) partner repository with the data set identifier PXD027754 (https://www.ebi.ac.uk/pride/archive/projects/PXD027754).

#### Statistics.

All cellular experiments were performed in at least 3 biologically independent experiments except for oxygraph experiments, which were performed in at least 6 biological replicates. Animal data were generated from 5 animals in each group. Data were expressed as mean ± SD unless specified, and groups were compared using 1-way ANOVA with Bonferroni’s post hoc analyses for quantitative data that were normally distributed and the Kruskal-Wallis test for skewed data. Details of the bioinformatics analyses, identifying temporal clusters, and integrating data sets are described in the section on unbiased approaches.

#### Study approval.

All studies were approved by the Institutional Animal Care and Use Committee at the Cleveland Clinic (2014-1351).

## Author contributions

A Kumar, NW, SM, AB, RSN, and GD were involved in design of experiments, analysis and interpretation of the data, initial manuscript writing, and review of the final manuscript. LL and BW performed the proteomics analyses. SSS, MS, A Kerr, AKC, JS, AHA, and CH assisted with analysis and interpretation of data, troubleshooting experiments, and editing the final manuscript. SD was involved in overall design, supervising all components of the study, troubleshooting experiments, analysis and interpretation of the data, writing the initial and final drafts, and obtaining funding. All authors have reviewed the final manuscript and approve the submission. Author order for equal contributors: The decision for the order of the authors was based on the conceptualization of the experiments, performing the animal and cellular studies, analysis of data, bioinformatics analyses and interpretation, and writing the initial draft of the manuscript and was done in discussion with both the equal contributing authors and the senior author.

## Supplementary Material

Supplemental data

Supplemental table 1

Supplemental table 2

Supplemental table 4

Supplemental table 5

Supplemental table 9

Supplemental table 10

## Version 1 12/22/2021

Electronic publication
