# Supplementary material for: Metabolic reprogramming during hyperammonemia targets mitochondrial function and postmitotic senescence
Source: JCI Insight. 2021 Dec 22;6(24):e154089. doi: 10.1172/jci.insight.154089 (PMC8783680; doi:10.1172/jci.insight.154089)
Supplement: Supplemental data [file jciinsight-6-154089-s052.pdf]

S.Fig 1. Isolated mitochondrial fraction purity. Immunoblot for voltage dependent anion channel (mitochondria), protein disulfide isomerase (PDI; Endoplasmic reticulum), pan cadherin (plasma membrane) and alpha-tubulin (cytoplasm) to demonstrate purity of cellular fractions. n=1

S.Fig 2. Isolated mitochondrial proteome identifies electron transport chain protein expression changes with several that are improved or partially with withdrawal. (A) Oxidative phosphorylation pathway with an overlay of log fold expression quantification of differentially expressed proteins (DEP) from the isolated mitochondrial proteome dataset from C2C12 myotubes treated with 10mM ammonium acetate for 24 h (Am) with 24 h of ammonia removal (WD) compared to the untreated (UnT) myotubes. (B) Protein heatmap of the same oxidative phosphorylation pathway for the following comparisons: Am vs UnT, WD vs Am, and WD vs UnT. Red= increased expression, green= decreased expression. DEP significance was taken at $p<0.05$. All experiments were performed in n=3 biological replicates. Significance was calculated using an unpaired Student's t-test.

S.Fig 3. Unsupervised clustering reveals unique groups of molecules and their relationships across treatments. Heatmap with hierarchical clustering of rows and columns for A. Whole cell proteomics, B. Mitochondrial proteomics, and C. RNAseq from myotubes that are either untreated (UnT), treated for 24h with 10mM ammonium acetate (AmAc), and treated for 24h with AmAc and then have withdrawal (WD) of AmAc by media replacement for the final 24h of treatment. Dimensional reduction was performed using a $p<0.05$ for proteomics, and adjusted p-value of 0.05 for RNAseq using unpaired Student's t-test following Benjamini Hochberg correction. All experiments were performed in n=3 biological replicates.

S.Fig 4. Hyperammonemia impairs respiration in mitochondria isolated from differentiated myotubes. Representative oxygraph tracings from isolated mitochondria from untreated (UnT) and 24 h of 10mM ammonium acetate (AmAc) treated differentiated myotubes. Oxygen consumption was measured in isolated mitochondria in respiration buffer in the basal state and in response to electron transport chain (ETC) complex substrates and inhibitors sequentially. After initial stabilization, 2 mM malate(M) and 2.5 mM pyruvate(P) were added. This was followed by 2.5 mM ADP(D); 10 mM glutamate(G); 10 mM succinate(S); 2 uM increments of

FCCP for measuring maximum respiration; 375 nM rotenone (Rot.) to inhibit Complex I; 125 nM antimycin A to inhibit Complex III; 2 mM ascorbate and 2 mM tetramethyl p-phenylene diamine to test complex IV activity; and 50 mM sodium azide to inhibit complex IV activity. R.R.; reserve respiratory capacity; Max. R.: maximum respiration. All data expressed as mean±SD from at least 4 sets of isolated mitochondria each group *p <0.05; **p <0.01; ***p <0.001 compared to untreated controls determined using unpaired Student's t-test.

S.Fig. 5. Ammonia rechallenge after withdrawal in C2C12 myotubes. (A) Representative photomicrographs and diameter of myotubes, expressed as a percentage of controls, that were either untreated, treated for 24h with ammonium acetate (AmAc), or following AmAc for 24h, ammonia withdrawal for 24h and subsequent rechallenge with AmAc for 24h (WD+Re.). Quantitative data are represented as box and whisker plots, with box bounds from 1st quartile to the 3rd quartile, median line, x as the mean, and whiskers ranging from minimum to maximum values, outliers represented as circles for myotube diameter as a percentage of controls. (B) Oxygen consumption measured by high resolution respirofluorometry in intact myotubes that have been treated with AmAc WD+Re. (C) ATP content of myotubes that were either untreated or treated with AmAc with or without WD+Re. (D) Representative immunoblots and densitometry for carbonylated proteins in untreated myotubes compared to those treated with AmAc or WD+Re. (E) Representative immunoblots and densitometry for p16INK and p21 in myotubes that were untreated, treated with AmAc or WD+Re. All data expressed as mean±SD from at least 6 biological replicates in oxygraph studies and at least 3 biological replicates in all other studies. *p<0.05; **p<0.01; ***p<0.001 using unpaired Student's t-test or one-way analysis of variance followed by Bonferroni post-hoc comparison tests.

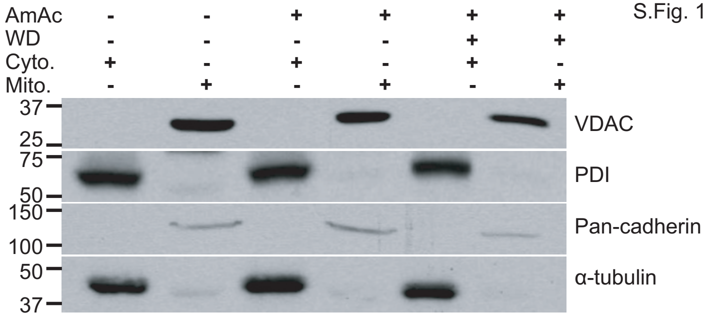

S.Fig 1. Isolated mitochondrial fraction purity. Immunoblot for voltage dependent anion channel (mitochondria), protein disulfide isomerase (PDI; Endoplasmic reticulum), pan cadherin (plasma membrane) and alpha-tubulin (cytoplasm) to demonstrate purity of cellular fractions. n=1

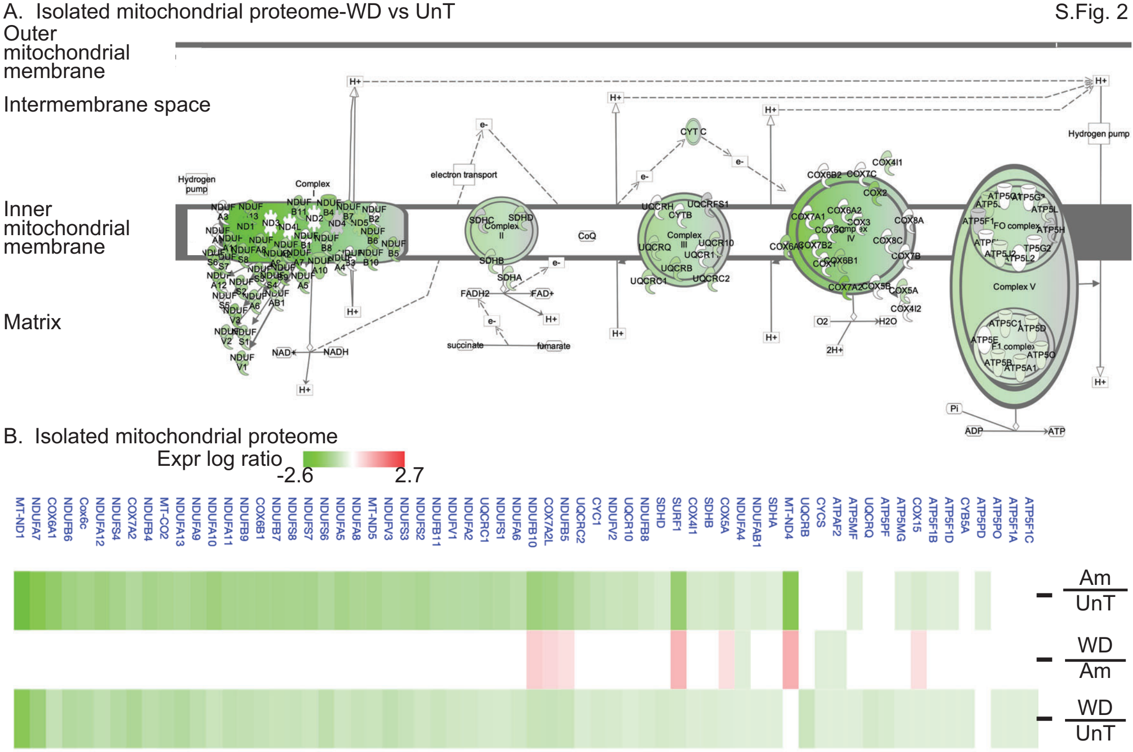

S. Fig. 2. Isolated mitochondrial proteome identifies electron transport chain protein expression changes with several that are improved or partially with withdrawal. (A) Oxidative phosphorylation pathway with an overlay of log fold expression quantification of differentially expressed proteins (DEP) from the isolated mitochondrial proteome dataset from C2C12 myotubes treated with 10mM ammonium acetate for 24 h (Am) with 24 h of ammonia removal (WD) compared to the untreated (UnT) myotubes. (B) Protein heatmap of the same oxidative phosphorylation pathway for the following comparisons: Am vs UnT, WD vs Am, and WD vs UnT. Red= increased expression, green= decreased expression. DEP significance was taken at $p<0.05$. All experiments were performed in n=3 biological replicates. Significance was calculated using an unpaired Student's t-test.

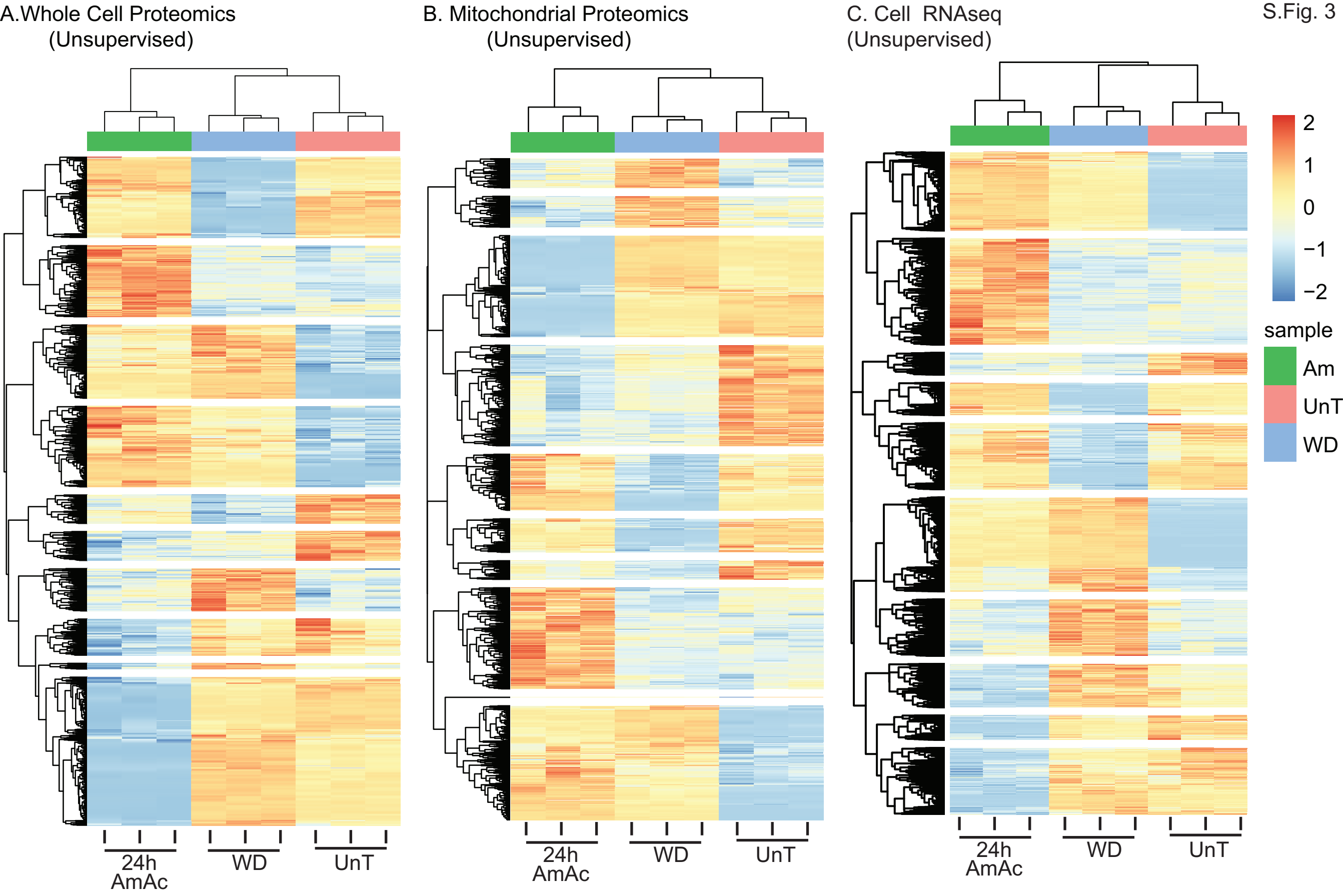

S.Fig 3. Unsupervised clustering reveals unique groups of molecules and their relationships across treatments. Heatmap with hierarchical clustering of rows and columns for (A) Whole cell proteomics, (B) Mitochondrial proteomics, and (C) RNAseq from myotubes that are either untreated (UnT), treated for 24h with 10mM ammonium acetate (AmAc), and treated for 24h with AmAc and then have withdrawal (WD) of AmAc by media replacement for the final 24h of treatment. Dimensional reduction was performed using a $p<0.05$ for proteomics, and adjusted p-value of 0.05 for RNAseq using unpaired Student's t-test following Benjamini Hochberg correction. All experiments were performed in n=3 biological replicates.

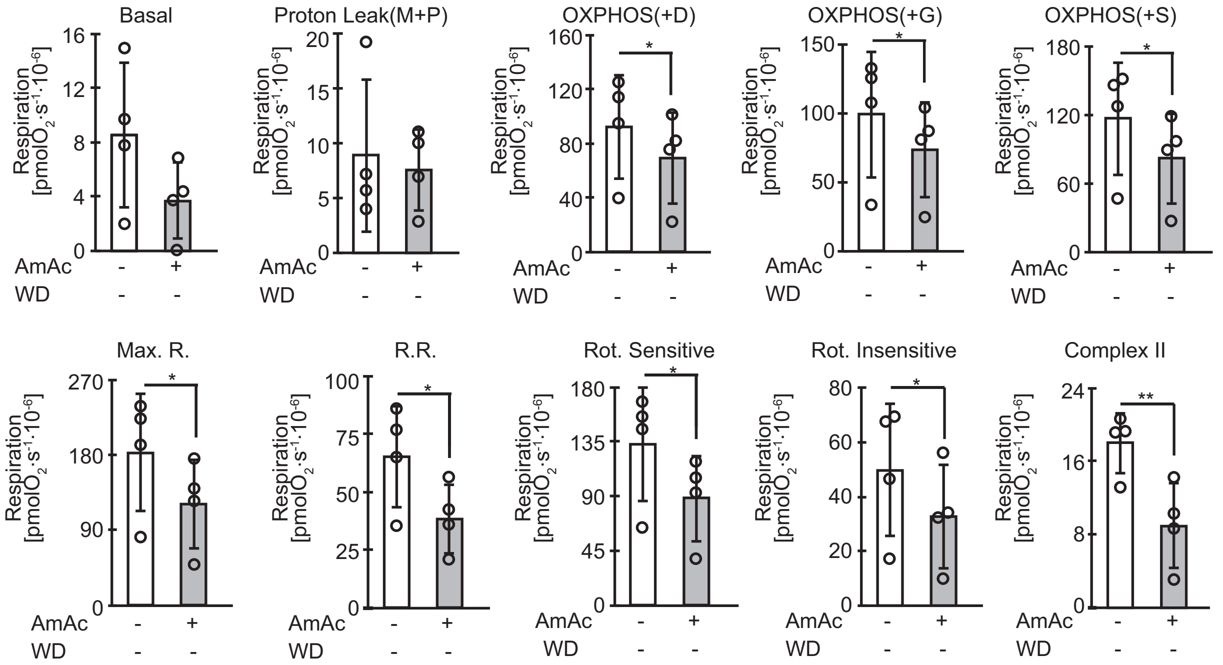

S.Fig. 4. Hyperammonemia impairs respiration in mitochondria isolated from differentiated myotubes. Representative oxygraph tracings from isolatedmitochondria from untreated (UnT) and 24 h of 10mM ammonium acetate (AmAc) treated differentiated myotubes. Oxygen consumption was measured in isolated mitochondria in respiration buffer in the basal state and in response to electron transport chain (ETC) complex substrates and inhibitors sequentially. After initial stabilization, 2 mM malate(M) and 2.5 mM pyruvate(P) were added. This was followed by 2.5 mM ADP(D); 10 mM glutamate(G); 10 mM succinate(S); 2 uM increments of FCCP for measuring maximum respiration; 375 nM rotenone (Rot.) to inhibit Complex I; 125 nM antimycin A to inhibit Complex III; 2 mM ascorbate and 2 mM tetramethyl p-phenylene diamine to test complex IV activity; and 50 mM sodium azide to inhibit complex IV activity. R.R.; reserve respiratory capacity; Max. R.: maximum respiration. All data expressed as mean±SD from at least 4 sets of isolated mitochondria each group *p<0.05; **p<0.01; ***p<0.001 compared to untreated controls determined using an unpaired Student’s t-test.

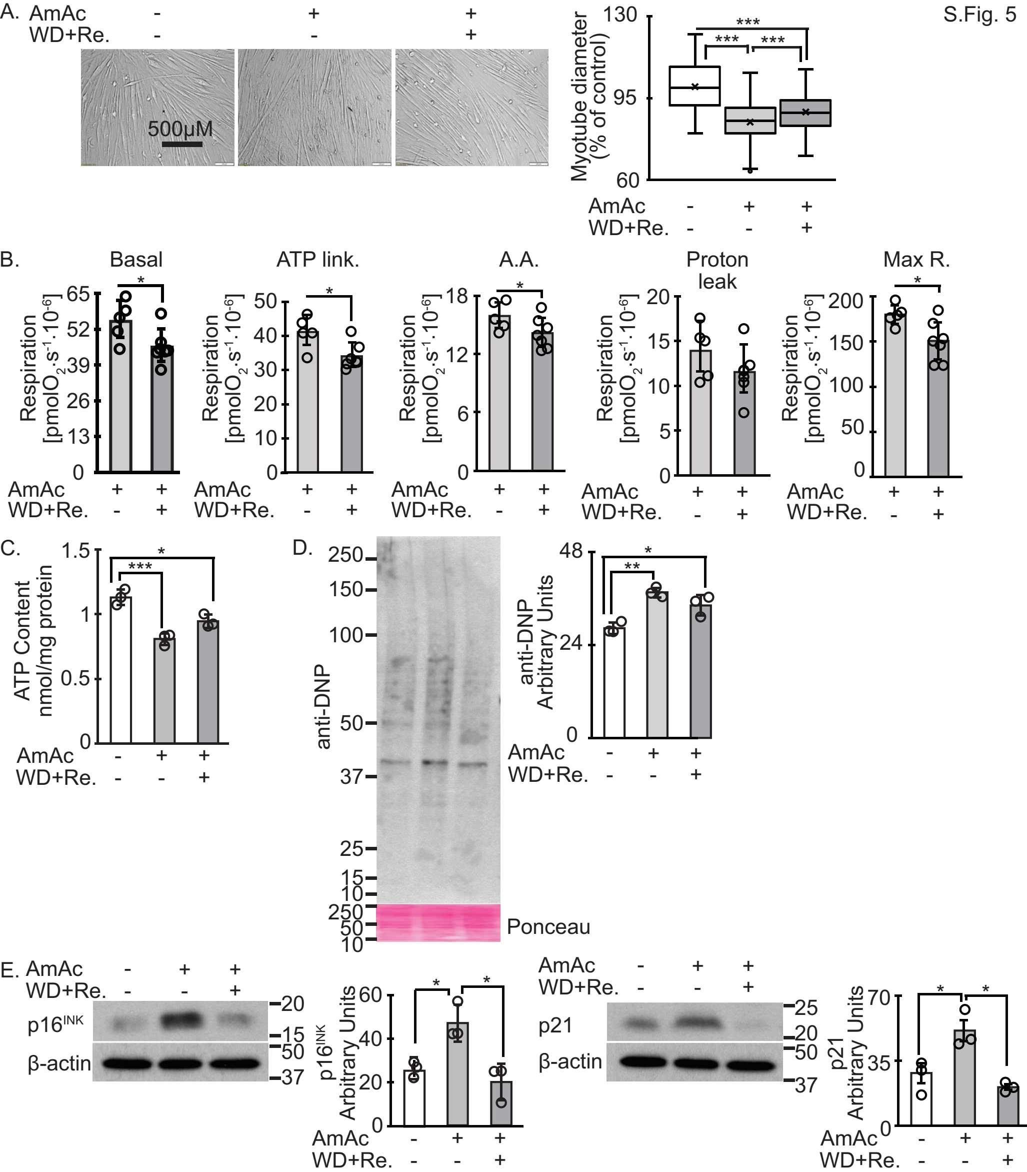

S.Fig. 5 Ammonia rechallenge after withdrawal in C2C12 myotubes. (A) Representative photomicrographs and diameter of myotubes, expressed as a percentage of controls, that were either untreated, treated for 24h with ammonium acetate (AmAc), or following AmAc for 24h, ammonia withdrawal for 24h and subsequent rechallenge with AmAc for 24h (WD+Re.). Quantitative data are represented as box and whisker plots, with box bounds from 1st quartile to the 3rd quartile, median line, x as the mean, and whiskers ranging from minimum to maximum values, outliers represented as circles for myotube diameter as a percentage of controls. (B) Oxygen consumption measured by high resolution respirofluorometry in intact myotubes that have been treated with AmAc WD+Re. (C) ATP content of myotubes that were either untreated or treated with AmAc with or without WD+Re. (D) Representative immunoblots and densitometry for carbonylated proteins in untreated myotubes compared to those treated with AmAc or WD+Re. (E) Representative immunoblots and densitometry for p16INK and p21 in myotubes that were untreated, treated with AmAc or WD+Re. All data expressed as mean±SD from at least 6 biological replicates in oxygraph studies and at least 3 biological replicates in all other studies. *p<0.05; **p<0.01; ***p<0.001 using unpaired Student's t-test or one-way analysis of variance followed by Bonferroni post-hoc comparison tests.

Supplementary Table 3. Electron transport chain proteins expressed in the cellular mitochondrial proteome.

Genes	AmAc vs UnT	WD vs AmAc	WD vs UnT	Complex	Complex I Module
MT-ND1	-2.37	N/A	-1.73	1	-
MT-ND4	-1.74	1.03	N/A	1	-
MT-ND5	-1.13	N/A	-0.68	1	-
NDUFA10	-1.32	N/A	-0.69	1	PP
NDUFA11	-1.16	N/A	-0.80	1	PP
NDUFA12	-1.32	N/A	-0.80	1	N/Q
NDUFA13	-1.06	N/A	-0.97	1	PP
NDUFA2	-0.92	N/A	-0.57	1	N
NDUFA4	-0.19	-0.20	-0.39	1	-
NDUFA5	-1.19	N/A	-0.63	1	Q
NDUFA6	-0.85	N/A	-0.59	1	Q
NDUFA7	-1.78	N/A	-1.37	1	Q
NDUFA8	-1.09	N/A	-0.73	1	PP
NDUFA9	-1.17	N/A	-0.83	1	Q
NDUFAB1	-0.45	N/A	-0.28	1	-
NDUFB10	-1.34	0.62	-0.71	1	PD
NDUFB11	-0.96	N/A	-0.67	1	PD
NDUFB4	-1.20	N/A	-0.84	1	PD
NDUFB5	-1.07	0.38	-0.69	1	PD
NDUFB6	-1.29	N/A	-0.97	1	PD
NDUFB7	-1.18	N/A	-0.72	1	PD
NDUFB8	-0.58	N/A	-0.48	1	PD
NDUFB9	-1.17	N/A	-0.78	1	PD
NDUFS1	-0.94	N/A	-0.50	1	N
NDUFS2	-1.09	N/A	-0.61	1	Q
NDUFS3	-1.04	N/A	-0.71	1	Q
NDUFS4	-1.19	N/A	-0.93	1	N
NDUFS6	-1.00	N/A	-0.82	1	N/Q
NDUFS7	-1.16	N/A	-0.66	1	Q
NDUFS8	-1.16	N/A	-0.73	1	Q
NDUFV1	-0.93	N/A	-0.60	1	N
NDUFV2	-0.69	N/A	-0.50	1	N
NDUFV3	-1.01	N/A	-0.79	1	PD
SDHA	-0.37	N/A	-0.36	2	-
SDHB	-0.50	N/A	-0.45	2	-
SDHD	-0.48	N/A	-0.52	2	-
CYC1	-0.73	N/A	-0.51	3	-
UQCR10	-0.71	N/A	-0.43	3	-
UQCRB	N/A	N/A	-0.64	3	-
UQCRC1	-0.97	N/A	-0.51	3	-
UQCRC2	-0.86	N/A	-0.46	3	-
UQCRQ	N/A	N/A	-0.52	3	-
COX15	-0.49	0.31	-0.19	4	-
COX4I1	-0.54	N/A	-0.42	4	-
COX5A	-0.57	0.15	-0.42	4	-
COX6A1	-1.53	N/A	-0.75	4	-
COX6B1	-1.17	N/A	-0.74	4	-
Cox6c	-1.21	N/A	-0.93	4	-
COX7A2	-1.00	N/A	-1.08	4	-
COX7A2L	-1.23	0.54	-0.70	4	-

CYB5A	N/A	N/A	-0.21	4	-
MT-CO2	-1.13	N/A	-0.91	4	-
SURF1	-1.44	0.95	-0.49	4	-
ATP5F1A	N/A	N/A	-0.15	5	-
ATP5F1B	-0.18	N/A	-0.14	5	-
ATP5F1C	N/A	N/A	-0.14	5	-
ATP5F1D	-0.12	N/A	-0.16	5	-
ATP5MF	-0.29	N/A	-0.24	5	-
ATP5MG	-0.23	N/A	-0.15	5	-
ATP5PD	-0.18	N/A	N/A	5	-
ATP5PF	N/A	N/A	-0.48	5	-
ATP5PO	N/A	N/A	-0.17	5	-
ATPAF2	N/A	-0.27	-0.29	5	-
CYCS	N/A	-0.32	-0.27	CytoChromeC	-

Numbers indicate log2 fold change of protein expression in isolated cellular mitochondrial untargeted quantitative proteomics; AmAc= ammonium acetate; UnT= untreated; WD= ammonia withdrawal; N/A = protein is not differentially expressed; N= dehydrogenase module (Complex I); N/Q= dehydrogenase module and/or hydrogenase module (Complex I); PD= ; PP= proton transloacation module (Complex I) ; Q= hydrogenase module (Complex I); . $p<0.05$ in n=3 biological replicates.

Supplementary Table 6. Key Reagents

REAGENT or RESOURCE	SOURCE	IDENTIFIER
**Antibodies** (clone number (experimental usage; dilution used))		
Mouse monoclonal anti-α-Tubulin (TU-02 (immunoblot normalization in isolated mitochondrial purity in UnT, AmAc, WD treated myotubes; 1:5000))	Santa Cruz Biotechnology, Dallas, Texas	Cat# sc-8035
Mouse monoclonal anti-β-Actin (C4 (immunoblot normalization and senescence associated beta-galactosidase activity in UnT, AmAc, WD treated myotubes and gastroc muscle Sham or PCA rats with or without LOLA-R; 1:5000))	Santa Cruz Biotechnology, Dallas, Texas	Cat# sc-47778
Mouse polyclonal anti-citrate synthase (densitometry of CS in UnT, AmAc, WD treated myotubes and gastroc muscle from Sham or PCA rats with or without LOLA-R; 1:5000)	ProteinTech, Rosemont, IL	Cat# 16131
Goat polyclonal anti-DNP (carbonylated in proteins in UnT, AmAc, WD treated myotubes and in gastroc muscle from Sham or PCA rats with or without LOLA-R 1:10,000)	Bethyl Laboratories Inc., Montgomery, TX	Cat# A150-117A
Rabbit monoclonal anti-p16 INK4A (D7C1M) (senescence associated beta-galactosidase activity in UnT, AmAc, WD treated myotubes and gastroc muscle from Sham or PCA rats with or without; 1:2000))	Cell Signaling Technology, Danvers, MA	Cat# 80772T
Rabbit polyclonal anti-p21 (senescence associated beta-galactosidase activity in UnT, AmAc, WD treated myotubes and gastroc muscle from Sham or PCA gastroc; 1:2000))	ProteinTech, Rosemont, IL	Cat# 10355-1-AP
Mouse monoclonal anti-p53 (1C12 (senescence associated beta-galactosidase activity in UnT, AmAc, WD treated myotubes; 1:2000))	Cell Signaling Technology, Danvers, MA	Cat# 2524s
Rabbit polyclonal anti-pan-cadherin (densitometry of isolated mitochondrial purity in UnT, AmAc, WD treated myotubes; 1:2000)	Cell Signaling Technology, Danvers, MA	Cat# 4068
Mouse monoclonal anti-PDI (RL90 (densitometry of isolated mitochondrial purity in UnT, AmAc, WD treated myotubes; 1:2000))	Novus Biologicals, Littleton, CO	Cat# NB300-517
Rabbit polyclonal anti-phospho-p53 (Ser15 (senescence associated beta-galactosidase activity in UnT, AmAc, WD treated myotubes and gastroc muscle from Sham or PCA rat with or without LOLA-R; 1:1000))	Cell Signaling Technology, Danvers, MA	Cat# 9284
Rabbit polyclonal anti-VDAC (isolated mitochondrial purity in UnT, AmAc, WD treated myotubes; densitometry of voltage dependent anion channel in UnT, AmAc, WD treated myotubes and gastroc muscle from Sham or PCA rats with or without LOLA-R; 1:2000)	Cell Signaling Technology, Danvers, MA	Cat# 4866
Goat anti-mouse IgG3, fc gamma Specific, HRP Conjugate (secondary antibody for anti-DNP; 1:5000)	Cell Signaling Technology, Danvers, MA	Cat# 75952
Anti-rabbit IgG, HRP-Linked (secondary antibody for anti-p16 INK4, anti-p21, anti-phospho-p53, and anti-pan-cadherin; 1:5000)	Cell Signaling Technology, Danvers, MA	Cat# 7074s

Anti-mouse IgG, HRP-Linked (secondary antibody for anti-p53, anti-citrate synthase, anti-PDI, anti-α-Tubulin, anti-β-actin; 1:5000)	Cell Signaling Technology, Danvers, MA	Cat# 7076s
**Chemicals, peptides, and recombinant proteins**		
α-Ketoglutaric acid	Sigma-Aldrich, St. Louis, MO	Cat# 75890
α-Ketoglutaric acid-$^{13}C_5$	Cambridge Isotopes, Tewksbury, MA	Cat# CLM-2411-PK
β-Mercaptoethanol	Sigma-Aldrich, St. Louis, MO	Cat# M3148
(+)-Sodium L-ascorbate (Ascorbate)	Sigma-Aldrich, St. Louis, MO	Cat# A7631
(L)-Malic Acid (Malate)	Sigma-Aldrich, St. Louis, MO	Cat# M1000
2,4'-Dinitrophenylhydrazine (DNPH)	Sigma-Aldrich, St. Louis, MO	Cat# D199303
2,7' -Dichloorofluorescin diacetate (DCFDA)	Sigma-Aldrich, St. Louis, MO	Cat# D6883
3-[(3-cholamidopropyl) dimethylammonio]-1-propanesulfonate (CHAPS)	EMD Millipore Corp., Billerica, MA	Cat# 220201
3,3' -Diaminobenzidine (DAB)	Sigma-Aldrich, St. Louis, MO	Cat# D8001
4-Methlyumbeliferyl β-D-galactopyranoside (MUG)	Sigma-Aldrich, St. Louis, MO	Cat# M1633
5-Bromo-4-Chloro-3-Indolyl β-D-Galactopyranoside (X-Gal)	ThermoFisher Scientific, Waltham, MA	Cat# B1690
Adenosine diphosphate (ADP)	Sigma-Aldrich, St. Louis, MO	Cat# A5285
Adenosine triphosphate (ATP)	Sigma-Aldrich, St. Louis, MO	Cat# A2383
Ammonium acetate	Sigma-Aldrich, St. Louis, MO	Cat# A7330
Antimycin a	Sigma-Aldrich, St. Louis, MO	Cat# A8674
Benzamidine	Honeywell Fluka Chemicals, Charlotte, NC	Cat# 12072
Bis-Tris	Sigma-Aldrich, St. Louis, MO	Cat# B4429
Carbonyl cyanide p-trifluoro-methoxyphenyl hydrazone (FCCP)	Sigma-Aldrich, St. Louis, MO	Cat# C2920
Citric acid	Sigma-Aldrich, St. Louis, MO	Cat# PHR1416
Citric acid	Sigma-Aldrich, St. Louis, MO	Cat# 251275
Citric acid-$^{13}C_6$	Cambridge Isotopes, Tewksbury, MA	Cat# CLM-9021-PK
Coomassie brilliant stain G-250	Bio-Rad Laboratories, Hercules, CA	Cat# 1610406

Coomassie brilliant stain R-250	Bio-Rad Laboratories, Hercules, CA	Cat# 1610400
Cytochrome c from bovine heart	Sigma-Aldrich, St. Louis, MO	Cat# C2037
Digitonin	Sigma-Aldrich, St. Louis, MO	Cat# D5628
Ethyl acetate	Fisher Scientific, Hampton. NH	Cat# E195SK-4
Fumaric acid	Sigma-Aldrich, St. Louis, MO	Cat# 47910
Fumaric acid-$^{13}C_4$	Cambridge Isotopes, Tewksbury, MA	Cat# CLM-1529-PK
Glutamate	Sigma-Aldrich, St. Louis, MO	Cat# G1626
Glycine	ThermoFisher Scientific, Waltham, MA	Cat# BP3815
L-Malic acid	Sigma-Aldrich, St. Louis, MO	Cat# 112577
L-Malic acid-$^{13}C_4$	Cambridge Isotopes, Tewksbury, MA	Cat# CLM-8065-PK
L-Ornithine L-Aspartate (LOLA)	Sigma-Aldrich, St. Louis, MO	Cat# O7125
Lead(II) nitrate	Sigma-Aldrich, St. Louis, MO	Cat# 228621
Magnesium chloride	Sigma-Aldrich, St. Louis, MO	Cat# 208337
Magnesium sulfate heptahydrate	Sigma-Aldrich, St. Louis, MO	Cat# M5921
MitoSOX™ red mitochondrial superoxide indicator	Invitrogen, ThermoFisher Scientific, Waltham, MA	Cat# M36008
N-Dodecyl β-D-maltoside 98%	Sigma-Aldrich, St. Louis, MO	Cat# D4641
N-tert-butyldimethylsilyl-N-methyltrifluroacetamide (MTBSTFA) and MTBSTFA + 1% TBDMCS (tert-butyldimethlysilyl ethers) sialylation reagent	ThermoFisher Scientific, Waltham, MA	Cat# TS-4890
N,N,N,N'-Tetramethyl-p-phenylenediamine dihydrochloride (TMPD)	Sigma-Aldrich, St. Louis, MO	Cat# T3134
NativePage™ 3 to 12%, bis-tris, 1.0 mm, mini gel	ThermoFisher Scientific, Waltham, MA	Cat# BN1001BOX
Nicotinamide adenine dinucleotide (NADH)	Sigma-Aldrich, St. Louis, MO	Cat# N6005
Nitro blue tetrazolium chloride (NBT)	ThermoFisher Scientific, Waltham, MA	Cat# N6495
Oligomycin	Sigma-Aldrich, St. Louis, MO	Cat# O4876
Phenylmethanesulfonylfluoride (PMSF)	Sigma-Aldrich, St. Louis, MO	Cat# P-7626

Potassium hexacyanoferrate (II) trihydrate	Sigma-Aldrich, St. Louis, MO	Cat# P3289
Potassium hexacyanoferrate (III)	Sigma-Aldrich, St. Louis, MO	Cat# P8131
Rifaximin	Sigma-Aldrich, St. Louis, MO	Cat# R9904
Rotenone	Sigma-Aldrich, St. Louis, MO	Cat# R8875
Sodium azide	Sigma-Aldrich, St. Louis, MO	Cat# S2002
Sodium chloride	Fisher Scientific, Hampton. NH	Cat#S640
Sodium phosphate	Sigma-Aldrich, St. Louis, MO	Cat# S5136
Sodium pyruvate (Pyruvate)	Sigma-Aldrich, St. Louis, MO	Cat# P2256
Sodium pyruvate-$^{13}C_3$	Cambridge Isotopes, Tewksbury, MA	Cat# CLM-2440-PK
Sodium succinate dibasic hexahydrate (Succinate)	Sigma-Aldrich, St. Louis, MO	Cat# S2378
Succinic acid	Sigma-Aldrich, St. Louis, MO	Cat# 398055
Succinic acid-$^{13}C_4$	Cambridge Isotopes, Tewksbury, MA	Cat# CLM-1371-PK
Tricine	Sigma-Aldrich, St. Louis, MO	Cat# T0377
**Critical Commercial Assays**		
ATP determination kit	Invitrogen, ThermoFisher Scientific, Waltham, MA	Cat# A22066
Lipid peroxidation (MDA) assay kit (Colorimetric/Fluorometric) (TBARS)	Abcam, Cambridge, UK	Cat# ab118970
MiR05-kit	O2k-Network Lab, Innsbruck, Austria	Cat# MiPNet22.10 MiR05-Kit
**Deposited data**		
Cellular proteomics	This paper	ProteomeXchange Consortium via the PRIDE partner repository with the dataset identifier PXD027754 and 10.6019/PXD027754
Mitochondrial proteomics	This paper	
Tissue proteomics	This paper	
Cellular RNAseq	This paper	https://github.com/dasaraslab/Unbiased
**Experimental models: cell lines**		
C2C12 myotubes	ATCC, Manassas, VA	Cat# CRL-1772
**Experimental models: organisms/strains**		
Rats: Sprague-Dawley	Charles River Laboratory, Wilmington, MA	Strain code# 400

Software and algorithms		
Adobe Illustrator 2021	Adobe, San Jose, CA	https://www.adobe.com/products/illustrator.html?sdid=KKQML&mv=search&ef_id=EAIaIQobChMIxbejkY7X8wIVy3xvBB1b7whaEAAYASAAEgKRYfD_BwE:G:s&s_kwcid=AL!3085!3!442365417815!e!!g!!adobe%20illustrator!1711729586!70905759510&gclid=EAIaIQobChMIxbejkY7X8wIVy3xvBB1b7whaEAAYASAAEgKRYfD_BwE
DatLab 6	Oroboros, Innsbruck, Austria	Cat# 27142-01
g:Profiler	Open Source	https://biit.cs.ut.ee/gprofiler/gost
ImageJ	NIH, Bethesda, MD	https://imagej.nih.gov/ij/
IPA	Qiagen, Hilden, Germany	https://digitalinsights.qiagen.com/products-overview/discovery-insights-portfolio/analysis-and-visualization/qiagen-ipa/
R studio	Open Source	https://www.rstudio.com/products/rstudio/download/

Supplementary Table 7. Clusters of changes in expression of genes and proteins during hyperammonemia and following ammonia withdrawal/lowering

Cluster	Name	Expression change from untreated with AmAc treatment	Expression change from untreated with WD
Progressive	a.	Increases	Increases further than the change with AmAc with WD
	j.	Decreases	Decreases further than the change with AmAc with WD
Persistent	b.	Increases	Increases with respect to UnT, but not significantly different compared to treatment with AmAc
	i.	Decreases	Decreases with respect to UnT, but not significantly different compared to treatment with AmAc
Partially reversed	d.	Increases	Increased with respect to UnT, but less than that seen with AmAc
	g.	Decreases	Decreased with respect to UnT, but less than that seen with AmAc
Completely Reversed	e.	Increases	No significant difference compared to UnT
	f.	Decreases	
Overcorrection	c.	Increases	Decreases
	h.	Decreases	Increases

AmAc: 24h 10mM ammonium acetate treatment; UnT: untreated; WD: Treatment with 24h 10mM ammonium acetate followed by 24h ammonium acetate withdrawal

Supplementary Table 8. Verified and non-verified mitochondrial proteins against MitoCarta3.0

	Whole Cell proteome	Mito proteome	Verified MitoCarta 3.0	Count	Rationale
Group 1	yes	yes	yes	356	All verified mitochondrial proteins found in the mitochondria also detected in the whole cell
Group 2	yes	no	yes	22	Mitochondrial targeted proteins detected in the whole cell that did not enter the mitochondria
Group 3	no	yes	yes	170	Mitochondrial proteins that are not concentrated enough to be present in the whole cell lysate
Group 4	no	no	yes	592	Verified mitochondrial proteins not detected in C2C12 cells or mitochondria
Group 5	yes	no	no	634	Non mitochondrial whole cell proteins
Group 6	no	yes	no	400	Non verified mitochondrial proteins OR transported proteins
Group 7	yes	yes	no	1197	Transported cytosolic or other non-mitochondrial proteins OR non-verified mitochondrial proteins

Mito: isolated mitochondrial proteome
